# Genomic Analysis of Localized High-Risk Prostate Cancer Circulating Tumor Cells at the Single-Cell Level

**DOI:** 10.3390/cells9081863

**Published:** 2020-08-08

**Authors:** Aline Rangel-Pozzo, Songyan Liu, Gabriel Wajnberg, Xuemei Wang, Rodney J. Ouellette, Geoffrey G. Hicks, Darrel Drachenberg, Sabine Mai

**Affiliations:** 1 Cell Biology, Research Institute of Hematology and Oncology, University of Manitoba, CancerCare Manitoba, Winnipeg, MB R3C 2B1, Canada; wellen68@gmail.com; 2 Department of Biochemistry and Medical Genetics, Research Institute of Hematology and Oncology, University of Manitoba, Winnipeg, MB R3C 2B1, Canada; Songyan.Liu@umanitoba.ca (S.L.); Geoff.Hicks@umanitoba.ca (G.G.H.); 3 Atlantic Cancer Research Institute, Pavillon Hôtel-Dieu, 35 Providence Street, Moncton, NB E1C 8X3, Canada; gabrielw@canceratl.ca (G.W.); rouellette@canceratl.ca (R.J.O.); 4 Manitoba Prostate Center, Cancer Care Manitoba, Section of Urology, Department of Surgery, University of Manitoba, Winnipeg, MB R3E 0V9, Canada; drach13@mymts.net

**Keywords:** localized high-risk prostate cancer, circulating tumor cells, three-dimensional (3-D) telomere profiling, laser microdissection, whole-exome genome sequencing, somatic single nucleotide variants, copy number alterations, precision medicine

## Abstract

Accurate risk classification of men with localized high-risk prostate cancer directly affects treatment management decisions and patient outcomes. A wide range of risk assessments and classifications are available. However, each one has significant limitations to distinguish between indolent and aggressive prostate cancers. Circulating tumor cells (CTCs) may provide an alternate additional source, beyond tissue biopsies, to enable individual patient-specific clinical assessment, simply because CTCs can reveal both tumor-derived and germline-specific genetic information more precisely than that gained from a single diagnostic biopsy. In this study, we combined a filtration-based CTC isolation technology with prostate cancer CTC immunophenotyping to identify prostate cancer CTCs. Next, we performed 3-D telomere profiling prior to laser microdissection and single-cell whole-exome sequencing (WES) of 21 CTCs and 4 lymphocytes derived from 10 localized high-risk prostate cancer patient samples. Localized high-risk prostate cancer patient CTCs present a high number of telomere signals with lower signal intensities (short telomeres). To capture the genetic diversity/heterogeneity of high-risk prostate cancer CTCs, we carried out whole-exome sequencing. We identified 202,241 single nucleotide variants (SNVs) and 137,407 insertion-deletions (indels), where less than 10% of these genetic variations were within coding regions. The genetic variation (SNVs + indels) and copy number alteration (CNAs) profiles were highly heterogeneous and intra-patient CTC variation was observed. The pathway enrichment analysis showed the presence of genetic variation in nine telomere maintenance pathways (patients 3, 5, 6, and 7), including an important gene for telomere maintenance called telomeric repeat-binding factor 2 (TRF2). Using the PharmGKB database, we identified nine genetic variations associated with response to docetaxel. A total of 48 SNVs can affect drug response for 24 known cancer drugs. Gene Set Enrichment Analysis (GSEA) (patients 1, 3, 6, and 8) identified the presence of CNAs in 11 different pathways, including the DNA damage repair (DDR) pathway. In conclusion, single-cell approaches (WES and 3-D telomere profiling) showed to be useful in unmasking CTC heterogeneity. DDR pathway mutations have been well-established as a target pathway for cancer therapy. However, the frequent CNA amplifications found in localized high-risk patients may play critical roles in the therapeutic resistance in prostate cancer.

## 1. Introduction

Prostate cancer is a heterogeneous disease with indolent and aggressive forms. Prostate cancer is the most commonly diagnosed type of cancer in men [1]. A wide range of risk assessments and classifications are available. However, each one has significant limitations to distinguish between indolent and aggressive prostate cancers [2]. Patients are categorized into aggressive and potentially lethal disease based on tumor (T) stage, Gleason grade, the number of cores with tumor in the diagnostic biopsy, prostate-specific antigen (PSA), and imaging [3]. For some men with the highest heterogeneity or widest range of outcomes, recommendations range from active surveillance to surgery, radiation, or androgen deprivation therapy [4,5,6,7]. At present, despite improvements in prostate cancer management, relapse is still reported in the order of 30% and about 10% with rapid disease progression [8]. In addition, changes in prostate-specific antigen (PSA) concentrations was shown to not be a reliable parameter to inform prognosis [8]. The ultimate consequence of imprecise clinical prognostic grouping is that some patients with indolent tumors are overtreated, while others with an aggressive tumor are undertreated.

Recent studies have shown that specific genetic variations and copy number alterations (CNAs) are associated with disease aggressiveness and prediction of post-radical prostatectomy biochemical recurrence [9,10,11,12,13]; patients with high-risk polyclonal tumors relapse more frequently after primary therapy [13]. The problematic aspect of applying this information into clinical care is the associated risks of biopsy sampling, as well as the extensive spatial heterogeneity of the multifocal tumors typically present at diagnosis [14,15].

Other recent studies have addressed the potential use of circulating tumor cells (CTCs) as an additional source, beyond tissue biopsies, for a patient’s clinical assessment. CTCs can reveal tumor-derived and germline genetic information with more precision than the information obtained from a single diagnostic biopsy [14,15]. The use of CTCs, as liquid biopsies, in prostate cancer provides the opportunity for multiple and minimally invasive sampling for disease monitoring, response to treatment, and molecular profiling of the disease [16,17,18]. CTCs isolated from blood samples have shown to be found in early stages of the disease as well as in localized high-risk prostate cancer; however, the clinical significance of this has not yet been established [19,20,21,22]. In order to use CTCs as a minimally invasive sampling of prostate cancer and as biomarkers for patient stratification and selection of targeted therapy, it is important to ensure efficient enrichment (isolation), detection (identification imaging), and characterization (molecular profiling) strategies [23,24,25,26,27]. The limitations of the Food and Drug Administration (FDA)-approved platform Epithelial cell adhesion molecule (EpCAM)-based capture assays for the detection and enumeration of CTCs stimulated the development of many other technologies, including size-based capture enrichment devices [28]. 

In this study, we combined a filtration-based CTC isolation technology with prostate cancer CTC immunophenotyping to identify the prostate cancer CTCs [29,30]. After identification, we performed 3-D telomere profiling prior to laser microdissection and single-cell whole-exome sequencing (WES) of 21 CTCs and 4 lymphocytes from 10 localized high-risk prostate cancer patients. Our goal was to identify unique and common single nucleotide variants (SNVs), insertion-deletions (indels) mutations, and copy number alterations (CNAs) that could be used to predict high-risk lethal prostate cancer and treatment response for patients with clinically localized high-risk prostate cancers. Three-dimensional telomere profiling was performed prior to single-cell sequencing in the same patient sample, since alterations in telomere biology are one of the earliest events in prostate cancer tumorigenesis that continue during tumor progression [29,30]. The ability of CTCs’ 3-D telomere profiling in displaying tumor cell-dependent alterations in telomere architecture and its role as an important structural indicator of genomic instability present in each tumor cell genome have appeared in previous studies [31,32,33,34,35].

## 2. Materials and Methods

### 2.1. Patient Samples

Ten treatment-naïve patients with confirmed localized high-risk prostate cancer Gleason 8 or 9 had their CTCs and/or lymphocytes collected and analyzed. This study was conducted between 2017 and 2019. The patient clinical characteristics are summarized in Supplementary Table S1. This study obtained University of Manitoba Ethics Board approval and informed consent (HS14085; H2011:336; CCMB RRIC number 59-2011).

### 2.2. Isolation of CTCs Using the ScreenCell Filtration Technique and Immunostaining

CTC isolation by size-based filtration and immunostaining was performed as previously reported [34]. All samples were processed within 2h. The CTCs were isolated from the blood of prostate cancer patients using Screen Cell filtration devices (ScreenCell, Sarcelles, France), according to the manufacturer’s instruction [28]. All prostate cancer CTCs were recognized with a combination of prostate cancer cell-specific antibodies. Anti-androgen receptor conjugated with Alexa Fluor 488 (AR Antibody (441): sc-7305, Santa Cruz Biotechnology, Dallas, Texas, EUA), anti-cytokeratin 8,18,19 antibodies (Anti-Cytokeratin 8 + 18 + 19 antibody—ab41825, abcam, Cambridge, United Kingdom), as well as a negative marker for prostate cancer CTCs, CD45 (Anti-CD45 antibody (ab10558), abcam, Cambridge, United Kingdom) for lymphocyte staining was used. Dried isolation supports (ISs) were stored at 4 °C or -20 °C prior to quantitative 3-D telomere fluorescent in situ hybridization and laser microdissection for single-cell isolation. Two ISs were collected per patient.

### 2.3. Co-Immuno Telomere Three-Dimensional Quantitative Fluorescent In Situ Hybridization (3-D-QFISH)

The 3-D-QFISH was performed as previously described [32,33,34,35]. Briefly, the ISs or filters were rehydrated with 1x PBS (phosphate-buffered saline) for 5 min followed by a 10-min fixation in 3.7% formaldehyde/1x PBS. The filters were blocked in 4%BSA/4×SSC blocking solution for 5 min, then incubated with primary antibody anti-AR (1:500 dilution), anti-Cytokeratin 8 + 18 + 19 antibody (1:200 dilution), and anti-CD45 antibody (1:100 dilution) for 45 min at 37 °C in a humidified atmosphere. Then, 1× PBS for 5 min (3×) were performed to wash away the extra unbound primary antibody. Incubation with secondary goat anti-mouse antibody (1:500 dilution, Alexa Fluor 680 (Cy 5.5) ThermoFisher Scientific, Waltham, MA, USA) and secondary goat anti-rabbit antibody (1:500 dilution, Alexa Fluor 647 (Cy5) ThermoFisher Scientific, Waltham, MA, USA) was followed for 30 min at 37 °C in a humidified atmosphere. Then, 1× PBS three times for 5 min washes were performed to wash away the extra unbound antibody. Filters were dehydrated in an ethanol series and air-dried. Cyan 3 (Cy3) telomere-specific peptide nucleic acid (PNA) probe (DAKO, Agilent Technologies, USA) was applied before denaturation at 80 °C for 3 min, hybridization for 2 h (h) at 30 °C. Filters were washed in 70% deionized formamide (Sigma-Aldrich, St. Louis, MI, USA) in 10 mM Tris pH 7.4 for 15 min. Filters were removed from the metal support ring using an 8-mm biopsy punch, placed on a new slide, DAPI (4′,6-diamidino-2-phenylindole), ThermoFisher Scientific, Waltham, MA, USA) counterstained, and mounted with Vectashield (Vector Laboratories, Burlington, ON, Canada) with a coverslip.

### 2.4. Imaging and Analysis

For each patient sample, 30 CTC nuclei were analyzed using TeloView^TM^ software [36] (used with the permission of Telo Genomics Corp Inc. Toronto, ON, Canada). Telomeres were imaged using fluorescence microscopy (Zeiss AxioImager Z1 microscope (Carl Zeiss, Toronto, ON, Canada) equipped with an AxioCam HRm camera, using a 63×/1.4 oil plan apochromat objective lens). The imaging software ZEN 2.3 software was used for image acquisition. Three-dimensional imaging of telomeres was performed using 40 *z*-stacks, each with a thickness of 0.2 μm (*z* plane). The sampling distance of the *x*- and *y*-planes was 102 nm. The exposure time for Cy3 (telomeres) was maintained at a constant 444.54 milliseconds, whereas that for FITC, Cy5, Cy5.5, and DAPI varied. An FITC filter was used to determine the presence of AR antibodies, Cy5.5 for anti-cytokeratin 8,18,19 antibodies and Cy5 for CD45 antibodies. Images were deconvolved using a constrained iterative algorithm [36] at the manual strength of 7 for CY3 and 6 for DAPI. Each cell was analyzed for the number of telomere signals per nucleus, intensity of signal, presence of telomere aggregates (two or more signals that cannot be resolved due to proximity and defined as a signal with an intensity above the standard deviation of signal intensity for that cell), *a/c* ratio, and nuclear volume. These measurements were determined for CTCs from each patient isolated at diagnosis. When cells are captured on the ScreenCell filtration device, they are flattened due to the mild vacuum applied during isolation [32]. Therefore, the nuclear volumes and *a/c* ratios discussed here can only be seen in a comparative manner (CTCs vs. CTCs) and do not represent absolute measurements.

### 2.5. Laser Microdissection and Whole-Exome Amplification

**Figure 1:**
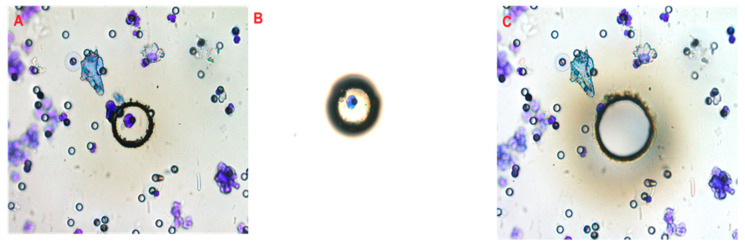
Principle of the laser capture microdissection. After circulating tumor cells (CTC) isolation, the CTCs were attached in a track-etched polycarbonate filter. The filter pores measure 6.5 ± 0.33 µm in diameter and retain 85–100% of tumor cells and only 0.1% of lymphocytes. (**A**) May-Gruenwald-Giemsa stain was performed on the filters for CTC identification by morphological and cytopathological criteria. Then, a UV laser beam was focused and used to cut a circle around the area of the target CTC or lymphocyte via an inverted microscope (Laser Microdissection Olympus IX microscope MMI CellCut—MMI GmbH—Molecular Machines & Industries, Eching, Germany). The dissected CTC was collected by photonic pressure using laser pressure to lift the dissected CTC into a collecting cap (**B**). The empty area that had contained the target cell can be visualized in **C**.

Prostate cancer CTCs and lymphocytes were isolated by laser microdissection. Giemsa (Millipore, Billerica, MA, USA) was used to stain the filters, allowing single CTCs and lymphocytes to be identified and isolated by Laser Microdissection Olympus IX microscope MMI CellCut (MMI GmbH—Molecular Machines & Industries, Eching, Germany) (Figure 1). Once isolated at the single-cell level, CTCs underwent whole-genome amplification (WES). The DNA of isolated CTCs and lymphocytes was amplified using the Ampli1™ WES kit (Menarini Silicon Biosystems, San Diego, CA, USA) according to the manufacturer’s instructions. Briefly, reactions conducted in the same tube followed these steps: Cell lysis, DNA digestion, ligation, and primary PCR according to the procedure of the supplier, resulting in a final volume of 50 µL of WES product. Genome integrity and quality were evaluated using the Ampli1™ QC kit (Menarini Silicon Biosystems San Diego, CA, USA) and PCR products were visualized via 1.5% agarose gel.

### 2.6. Whole-Exome Sequencing and Bioinformatics Analysis

DNA fragments of 180–280 bp in length were generated by a hydrodynamic shearing system (Covaris, MA, USA) with 1.0 μg of genomic DNA per sample. Remaining overhangs were converted into blunt ends via exonuclease/polymerase activities and enzymes from a TruSeq preparation kit were removed. After adenylation of the 3’ ends of DNA fragments, adapter oligonucleotides (TruSeq adaptors) were ligated. DNA fragments with ligated adapter molecules on both ends were selectively enriched in a PCR reaction. The PCR products were purified using an AMPure XP system (Beckman Coulter, Beverly, MA, USA) and quantified using the Agilent high sensitivity DNA assay on the Agilent Bioanalyzer 2100 system. The fragmented sequences were hybridized with probes using an Agilent SureSelect Human All Exon kit (Agilent Technologies, CA, USA). The clustering of the index-coded samples was performed on a cBot Cluster Generation System using a TruSeq PE Cluster Kit v4-cBot-HS (Illumina, San Diego, CA, USA) according to the manufacturer’s instructions. After cluster generation, the output was loaded into a chip and sequenced with the HiSeq X sequencer (Illumina, San Diego, CA, USA). A total of 21 CTCs and 4 lymphocytes were sequenced (Table 1). The number of CTC and or lymphocyte analysed per patient is shown in the Supplementary Materials Table S1.

The sequencing experiment produced raw fastq files (sequencing data available at SRA database, SRA accession: PRJNA633995; Temporary Submission ID: SUB7472754), which were preprocessed with Trim Galore (version 0.3.7) [37] in order to remove adapters and perform quality trimming. The mapping was performed with the Burrows-Wheeler Aligner (BWA) [38], more precisely the bwa-mem (version 0.7.8) algorithm, to the human reference genome (GRCh37 + decoy). The aligned bam file was processed using Samtools (version 1.0) [39] and Picard (version 1.111) [40]. SNV and indels calling were performed with GATK (version 3.1) [41] and respective annotation with Ensemble Effect Variant Predictor (VEP) web-version [42] using dbSNP (build 144) [43]. The copy number variations were identified using Control-FREEC (version 6.7) [44] and the genomic amplification was calculated between each CTC generating a file with merged SNVs from all lymphocytes. For the SNV and indel cutoff, we discarded variants with a mapping quality lower than 30 and read depth lower than 100. For the CNV calling, we considered only CNVs with gains above 2 copies. The SNVs and indels identified in the lymphocytes were used only to filter out variations not associated with the cancer genotype. For example, in the cluster analyses, we removed every SNV or indels also present in at least one lymphocyte. For CNV analysis, lymphocytes were used to set out the number of gains of copies in the CTCs. Gene set enrichment analysis (GSEA) was performed with EnrichR [45]. To compare our findings with known pathways and terms, we used KEGG [46], Gene Ontology [47], and Reactome [48] databases. We also used the PharmKGB database to associate genetic variations to known cancer drugs’ effect [49].

To reduce the probability of false positives, we selected the SNVs and indels with genotype quality ≥ 30 and reading depth ≥ 100 from all samples to be entered into a customized database (MySQL). We filtered out all SNVs and indels found in any of the lymphocyte samples in order to analyze only somatic variants.

## 3. Results

### 3.1. High-Risk Prostate Cancer CTCs Were Selected Based on Their Positive Staining for the Androgen Receptor and Cytokeratin 8, 18, and 19 and Negativity for CD45.

**Figure 2:**
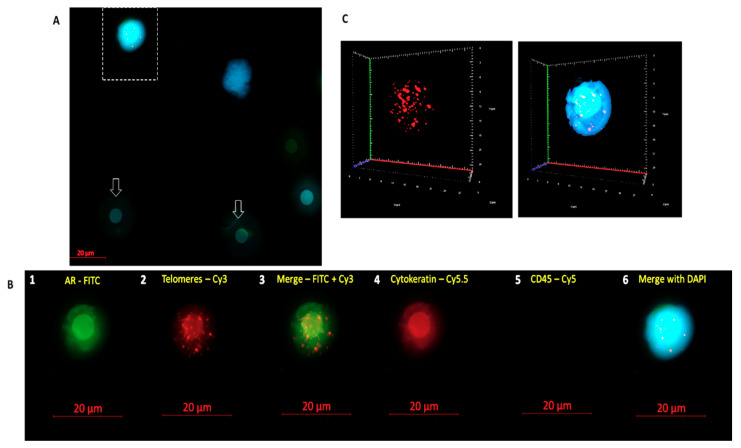
Example of a circulating tumor cell from a high-risk localized prostate cancer patient captured on top of a filter pore (**A**). The arrows show empty filter pores in A. Prostate cancer CTCs are recognized based on their androgen receptor (AR) and cytokeratin-positive staining and -negative staining of CD45 (**B**). (B1) Two-dimensional image showing a CTC AR positive in fluorescein isothiocyanate (FITC—green); (B2) CTC with the telomeres labeled with Cy3-labeled probe (red); (B3) Merge between FITC and telomeres; (B4) CTC cytokeratin positive in Cy5.5 (red); (B5) CTC CD45 negative; and (B6) merge of CTC counterstained with 4′,6-diamidino-2-phenylindole (DAPI) in blue. In (**C**), the same cell is shown in three-dimensional representation. Red spots represent telomere signals; and the blue is DAPI.

The study involved 10 patients with high-risk localized prostate cancer, aged 55–80 years (median, 75 years) at diagnosis. Their median PSA level and Gleason score were 6.4 ng/mL and 9, respectively. A summary of the clinical features of all 10 patients at baseline is shown in Supplementary Table S1. CTCs were collected at diagnosis and prior to treatment, using a size-based filtration technique (ScreenCell) [28]. We identified the prostate cancer CTCs based on their positive immunostaining for androgen receptor (AR) and cytokeratin 8, 18, and 19 (Cy) and negative staining for CD45 (Figure 2). Figure 2B shows an isolated CTC stained with AR antibody conjugated with Alexa Fluor 488 in green and Cytokeratin in red. AR stains both the intracytoplasmic region and the cell membrane, while cytokeratin identifies CTCs with epithelial origin.

### 3.2. CTCs from Localized High-Risk Prostate Patients Showed Telomere-Related Heterogeneity at the Single-Cell Level

**Figure 3:**
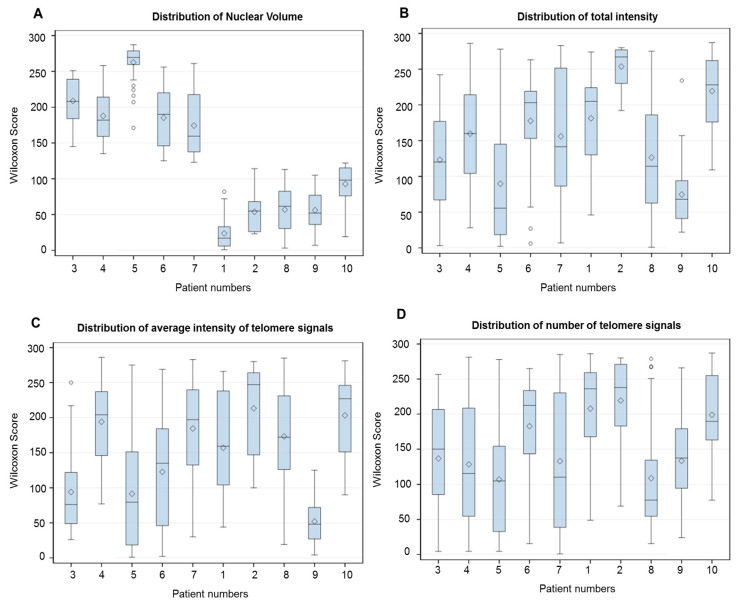

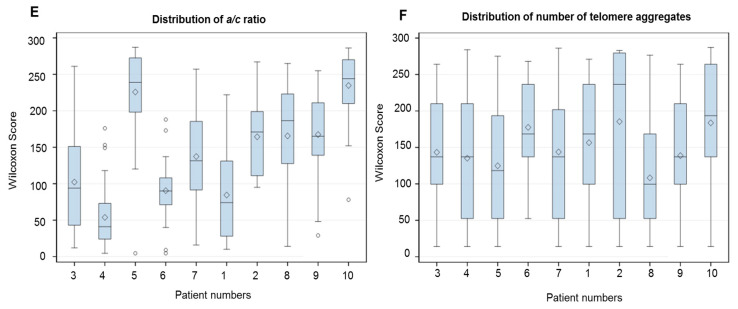
Representative bar plots to illustrate the intra and inter-sample variability of all telomere parameters. (**A**) Nuclear volume. (**B**) Total telomere signal intensity. (**C**) Average intensity (proportional of telomere length). (**D**) Total number of telomere signals. (**E**) a/c ratio (see material and methods). (**F**) Total number of telomere aggregates (see material and methods). The x-axis assigns one box for the CTC population analyzed per patient. The y-axis refers to output from Kruskal–Wallis test represented as Wilcoxon mean scores (determined using SAS software). Whiskers show minimum and maximum values, boxes represent 25–75% data ranges, horizontal lines within boxes are medians, and diamond symbols are means.

The three-dimensional telomere profile was used to adequately characterize prostate cancer CTCs and possible CTCs subpopulations. The TeloView^®^ (Telo Genomics, Toronto, ON, Canada) program provides information on the average intensity/telomere length distributions, which can be related to the clonality. We used the Wilcoxon score to explore the cell distribution of all TeloView^®^ parameters obtained for the 30 readings in each patient (Figure 3). At the individual patient level, it was apparent that CTC telomeres displayed considerable length heterogeneity (Figure 3B,C). The same was observed for the total number of telomere signals and *a/c* ratio (Figure 3D,E). The *a/c* ratio is correlated with cell cycle phase; a higher *a/c* ratio corresponds to the telomeres becoming organized in a disk-like formation in preparation for mitosis (later stages in the cell cycle) [36]. However, for the number of telomere aggregates and nuclear volume, the heterogeneity among CTCs were less evident. While all patients exhibited a high number of telomere aggregates (Figure 3F), the patients were dispersed in two subpopulations for nuclear volume (Figure 3A), based on the clear distinction of samples provided by this parameter. The telomere parameters were similar to those previously described by our group for high-risk localized prostate patients [30]. The lymphocytes’ telomere parameter distribution for each patient is provided in the Supplementary Materials Figure S1.

### 3.3. Whole-Exome Sequencing Showed Genetic Variation (SNVs and Indels) Associated with Telomere Maintenance Genes, Prostate Cancer, and Known Cancer Drug Response

We first analyzed the distribution of SNVs and indels among CTCs and lymphocytes. The genetic variation analysis (SNVs and indels) of single CTCs can detect important somatic mutations at diagnosis and new alterations acquired during the disease evolution or after treatment. Twenty-one single CTCs and 4 single lymphocytes DNA from 10 different patients were isolated and sequenced. We identified a total of 202,241 SNVs and 137,407 indels where less than 10% of these genetic variations were within coding regions (Table 2). We used the term genetic variations for the sum of SNVs and indels. Table 3 shows the number of SNVs and indels found in each CTC. As demonstrated in Table 3, each CTC showed a different number of SNVs and indels alterations. The lowest count of genetic variation (SNVs + indels) were found in the CTC sample 902_4 of patient 8 with 982 total and the highest number was found in the CTC sample 3013 of patient 2 with a total of 137.854 affected genes. No common genetic variation (SNVs + indels) was found in all 21 CTCs. However, we found common SNVs and indels in all patients in at least one of the CTCs. They all presented a deletion of four nucleotides (AAAG) in the *ITSN1* (Intersectin 1 gene) (rs71322246) and four SNVs in *PDE4DIP* (phosphodiesterase 4D interacting protein gene) (A/G, rs4997150) and (G/T, rs4997149); gene *ITSN1* (A/G, rs10222139); and *RCF1* (Respiratory supercomplex factor 1 gene) (A/G, rs2306596).

**Figure 4:**
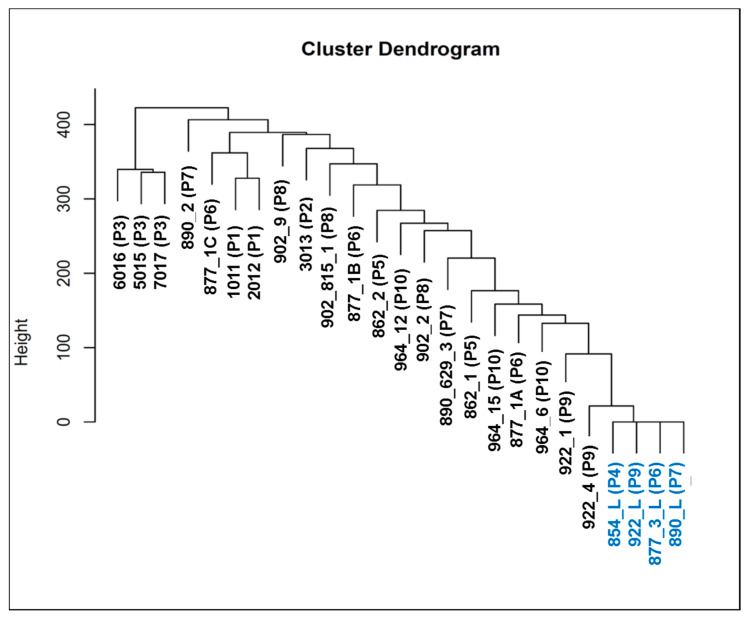
Hierarchical clustering of 21 CTCs (in black) and 4 lymphocytes (in blue) used in the analysis. The SNVs and indels are clustered using the average method, which performs a hierarchical cluster analysis using a set of dissimilarities between the samples. The y-axis (the heigh) are values of the distance in which two groups can split or merge, using the calculation of the Euclidean distance. P = patient.

Hierarchical clustering analysis was performed to compare patterns of SNVs and indels between the samples. Closer clusters in the dendrogram have more similar genetic variations than distant ones. As shown in Figure 4, all lymphocytes sit in the same cluster, which highlights the similarities between them. The lymphocyte cluster is very different from the cluster formed by CTCs samples 6016, 5015, and 7017, which is located in the opposite site of the dendrogram.

**Figure 5:**
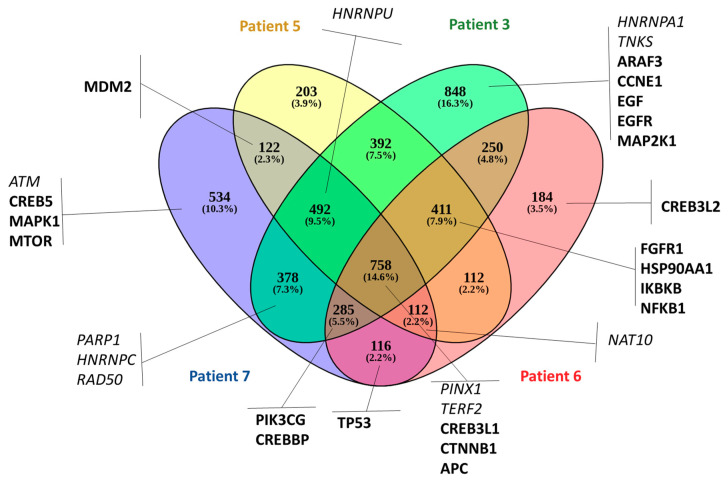
CTC-shared SNVs and indels for four prostate cancer patients. Venn diagram of genes with genetic variation within the coding sequence (frameshift indels and missense SNVs) in patient 3 (green), patient 5 (yellow), patient 6 (red), and patient 7 (blue). The genes highlighted in italic are from the GO term telomere maintenance (GO:0000723) and in bold from the KEGG related to prostate cancer (hsa05215).

Next, we evaluated the SNVs and indels in coding regions to highlight the presence of clinically relevant mutations. Patients 3, 5, 6, and 7 presented the highest number of genes among all patients, with genetic variation in coding regions (frameshift indels and/or missense SNVs). In Figure 5, we used a Venn diagram to illustrate the CTC-shared SNVs and indels between those four prostate cancer patients. We combined all genetic variation found in different CTCs isolated from the same patient. Patients 3, 5, 6, and 7 shared 758 genetic variations. To further understand the potential biological functions of CTC-shared SNVs and indels, we performed KEGG pathway (http://www.kegg.jp/) and GO (http://www.geneontology.org/) biological process analyses. In the total affected genes (4698), nine are associated with telomere maintenance (Gene Ontology term, GO:0000723) (*ATM*, *PARP1*, *HNRNPC*, *RAD50*, *PINX1*, *TERF2*, *NAT10*, *HNRNPA1*, and *TNKS*) (9 of 36 genes found in this pathway) and 18 genes were related to prostate cancer in the KEGG database (hsa05215) (18/89 found associated with prostate cancer) (Figure 5 in bold). In Supplementary Materials Figure S2, we illustrate the CTC-shared SNVs and indels results found for each patient and important genes affected.

**Figure 6:**
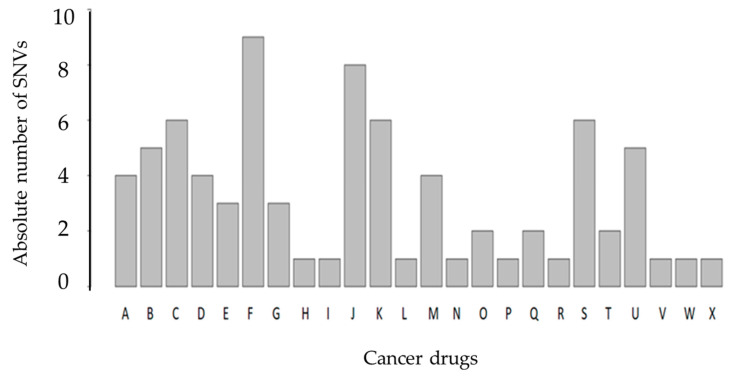
Bar plot of SNVs associated with known cancer drugs in the PharmGKB database. The PharmGKB database gathers all currently reported variant–drug interactions by at least two different scientific publications. The x-axis illustrates the cancer drugs, anthracyclines (**A**), capecitabine (**B**), carboplatin (**C**), cisplatin (**D**), cyclophosphamide (**E**), docetaxel (**F**), doxorubicin (**G**), doxorubicinol (**H**), exemestane (**I**), fluorouracil (**J**), gemcitabine (**K**), imatinib (**L**), irinotecan (**M**), letrozole (**N**), leucovorin (**O**), lonafarnib (**P**), methotrexate (**Q**), oxaliplatin (**R**), paclitaxel (**S**), platin compounds (**T**), thalidomide (**U**), tipiracil HCL (**V**), trastuzumab (**W**), and trifluridine (**X**). The y-axis is the number of SNVs found associated with each drug. The same SNV can affect the response of different drugs.

Lastly, we assessed the impact of SNVs found in all CTCs (10 patients) on known drug response targets according to the PharmGKB database. We identified nine genetic variations associated with response to docetaxel. In Figure 6, we show the number of SNVs associated with drug response. A total of 48 SNVs can affect drug response for 24 known cancer drugs. The 48 SNVs were identified in at least one CTC (Figure 6). To better contextualize Figure 6, we included a Supplementary Materials Table S2, where we list the number of SNVs found in our cohort associated with different cancer drug response. The percentage of our findings in all described SNVs associated with the same drug in the PharmKGB database and the patients where at least one SNV was found are shown in the sequential columns (Supplementary Materials Table S2). We used the Fisher exact test to perform an enrichment analysis of each drug in which we found correlated SNVs using the PharmGKB database. In summary, we checked if the group of SNVs previously identified had enough overrepresentation within a certain drug in the total all cancer drugs listed in the PharmGKB database and identified the following three drugs (*p*-value < 0.05): Cyclophosphamide, Docetaxel, and Thalidomide. These are the main drugs used in prostate cancer therapy (highlighted in blue in Supplementary Materials Table S2). The false discovery rate (FDR) using Benjamini–Hochberg correction was applied (threshold of 0.05) to the list of drugs and these three drugs remained the ones showing statistical significance.

### 3.4. Copy Number Alterations Identify Gene Amplifications Associated with High-Risk Prostate CTCs

**Figure 7:**
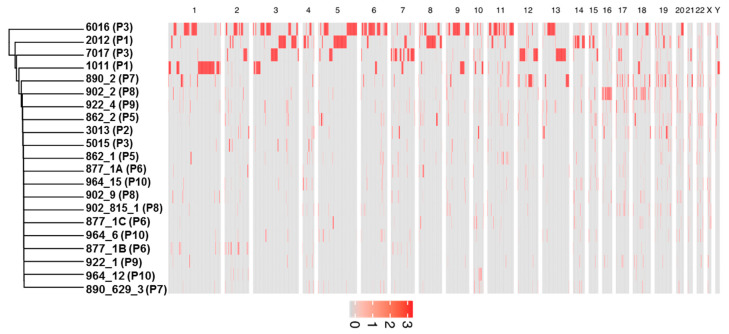
Heatmap showing significant large-scale amplification events in CTCs per whole chromosome. In this heat map, the chromosome number are arranged from left top to right, and 21 CTCs analysed flow vertical, top to bottom. Significant genomic amplifications are represented in red and the red intensity can vary according to the number of copies amplified. P = patient.

Somatic copy number alterations (CNAs) have an important role in genome instability and tumorigenesis. In contrast to SNVs and indels, which show substantial cell-to-cell heterogeneity, CNAs seem to exhibit genomic homogeneity in their patterns [51]. Genomic analyses of CTCs are crucial for understanding the underlying mechanisms required for cancer metastasis, including escape from the primary tumor site, entry in peripheral blood, and survival in the circulation [51]. To reduce the number of false negative genes for CNAs due to the procedure of single cells’ DNA amplification, we focused only on gene amplification. We noticed that significant portions of chromosomes in different samples, such as chromosome 1 in 1011; chromosomes 6 and 11 in 6016; chromosome 5 in 6016 and 2012; chromosome 7 in 2012; and chromosome 13 in 6016 and 7017 (Figure 7), were amplified. No common CNAs were found in all 21 CTCs or in all patients (found in at least one CTC).

**Figure 8:**
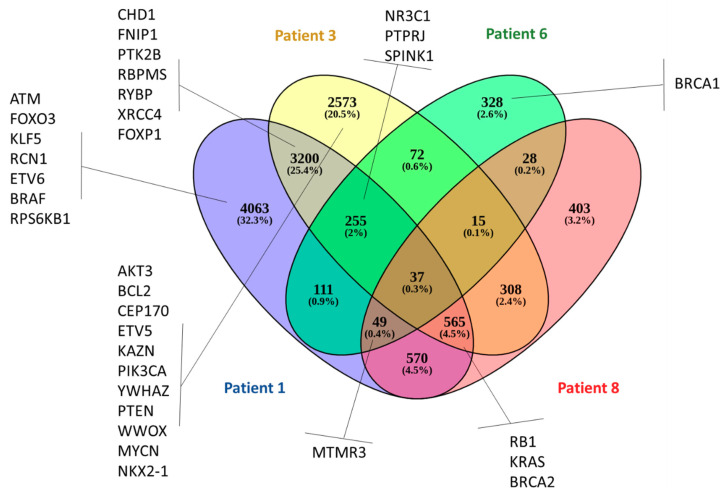
Venn diagram representing genes with amplification in at least one copy in patients 1 (blue), 3 (yellow), 6 (green), and 8 (red). The genes highlightened are similar genes’ amplification found in a previous study using CTCs from clinically localized high-risk prostate cancer (Friedlander et al. 2019) [52]. Some of the affected genes were also found by Ikeda et al. 2019 [53]. The list with the 37 commonly amplified genes is shown in Supplementary Materials Table S3.

Four of the nine patients analyzed had the highest number of amplifications (patients 1, 3, 6, and 8). The CTC-shared CNAs for those four patients are shown in Figure 8. Thirty-three amplifications have already been described as being associated with high-risk prostate and they are highlighted in Figure 8 [52,53]. In addition, 37 amplified genes were identified to be commonly shared by those four patients (Supplementary Materials Table S3). In order to understand how those 37 shared amplified genes are conected in known biological pathways, we next performed Gene Set Enrichment Analysis (GSEA) in the Reactome database (Table 4). The GSEA revealed that Poly(ADP-Ribose) Polymerase 1 (PARP1) amplification can affect three important DNA damage repair (DDR) pathways: Single strand break repair (SSBR), base excision repair (BER), and nucleotide excision repair (NER) [54]. In addition, USP21 amplification/ overexpression was positively correlated with human pancreatic ductal adenocarcinoma disease progression. USP21 promotes cell proliferation, tumor progression, and colony formation, and enhances cancer stem cell self-renewal. USP21 stabilizes the Wnt (Wingless-related integration site) pathway transcription factor 7 (TCF7) to activate gene expression in the Wnt network [55]. 

## 4. Discussion

Accurate risk classification of men with localized high-risk prostate cancer directly affects treatment management decisions and patient outcomes [2]. A wide range of risk assessment methods is available, each with significant limitations in discriminating between indolent and aggressive prostate cancers [6]. Sampling error, due to tumor multifocality tumors, failure of currently available imaging modalities to detect and assess local disease burden, and low-volume metastatic disease, can also increase the changes of misclassification [9]. Studies have shown that specific genetic alterations, such as mutations and copy number alterations, are associated with disease aggressiveness [9,10,11]. In addition, prostate patients with polyclonal tumors (and distinct mutational signatures) also relapse more frequently after primary therapy [13]. The main problem to apply this information into clinical care is the risk associated with biopsy sampling and the extensive spatial heterogeneity of the multifocal tumors, typically present at diagnosis. Repeated biopsy sampling can lead to infectious complications and even death [14]. CTCs have shown great clinical utility to characterize the genetic landscape of underlying tumors in prostate cancer and other solid tumors [34,35,36]. Obtaining the molecular profiles from patients with clinically localized disease may reduce the risk of misclassification and increase the detection of aggressive/lethal disease in need of immediate treatment. In addition, tumor cell-dependent alterations in telomere architecture represent a structural indicator of genomic instability present in prostate cancer CTCs [34,35,36]. The combination of telomere-related genomic instability with novel blood-based molecular profiling technologies, such as single-cell whole-exome sequencing, can improve our ability to monitor clonal evolution during therapy and disease progression.

Here, we performed 3-D telomere profiling prior to laser microdissection and single-cell whole-exome sequencing in localized high-risk prostate cancer patient samples. Our telomere measurements using TeloView^®^ showed that CTC telomeres displayed considerable length heterogeneity as well as the total number of telomere signals and *a*/*c* ratio, in agreement with our previously published results [35]. The CTCs of localized high-risk prostate cancer patients present a higher number of telomere signals than normal lymphocytes, with lower signal intensities (length), which signal an increase in telomere-related genomic instability [30]. We could see clearly that the nuclear volume measurements identified two subpopulations: Subpopulation 1 (including patients 3, 4, 5, 6, and 7) and subpopulation 2 (including patients 1, 2, 8, 9, and 10) (Figure 3A). We then used WES of single CTCs in order to detect the presence of multiple mutations within the same cell and further investigated tumor heterogeneity. In total, 21 single CTCs and 4 single lymphocytes from 10 different patients were isolated and sequenced. We identified a total of 202,241 SNVs and 137,407 indels where less than 10% of these genetic variations were within coding regions (Table 2). Since many regions of noncoding DNA play a role in the control of gene activity, it is possible that the number of genetic variations identified in noncoding regions is affecting the expression of a variate of genes. In addition, indels that can lead to frameshifts are usually under negative selection pressure [56]. Indels are the second most frequent type of genetic variation, followed by single nucleotide variations, and account for almost a quarter of the genetic variation implicated in human diseases [57]. We identified that the genetic variations (SFNVs + indels) and CNAs profiles were highly heterogeneous. Intra-patient CTC variation was observed for both SNVs + indels and CNAs (Figure 5 and Figure 8, and Supplementary Materials Figure S2). However, in reality, all 21 CTCs lacked common genetic variations (SNPs + indels) or CNAs, which is an indication of an extremely heterogeneous disease. In fact, localized prostate cancers are known to be genetically variable and frequently multifocal with multiple independently evolving clones [11]. To date, there is no understanding of whether this genetic variability can aid in management decisions for patient care. However, all patients presented a deletion of four nucleotides (AAAG) in the ITSN1 (intersectin 1 gene). ITSN1 inhibition is associated with cell proliferation and cell apoptosis inhibition. The ITSN1 gene is being considered a key biological target candidate for breast cancer [58]. The importance of ITSN1 deletion in prostate cancer still awaits future studies. We also found, in all patients, SNPs in the PDE4DIP and RCF1 genes. PDE4DIP (also known as myomegalin, MMGL) is a tumor marker for diagnosis and prognosis in patients with esophageal squamous cell carcinoma [59]. RCF1 is a member of the conserved hypoxia-induced gene 1 (Hig1) protein family [60]. The role of PDE4DIP and RCF1 genes in PC still awaits full investigation.

To explore the biological significance of genetic variants found in prostate cancer CTCs, we performed pathway enrichment analysis of the affected genes. Patients 3, 5, 6, and 7 showed 758 commonly genetic variations, where 9 telomere maintenance pathways are affected. This includes an important gene for telomere maintenance, called telomeric repeat-binding factor 2 (TERF2, also known as TRF2), which is one of the critical members of the shelterin complex. Loss or mutation of TRF2 results in telomere shortening, DNA damage, senescence, or apoptosis [61]. Alterations in TERF2 could explaining the increased telomere-related genomic instability in patients 3, 5, 6, and 7. A key opportunity arising from whole-exome sequencing analysis is the early identification of the patient’s drug response. To this end, we used the PharmGKB database to investigate the impact of the SNVs found in all CTCs on drug response. We identified nine genetic variations associated with response to docetaxel. Adjuvant docetaxel-based chemotherapy improved the overall survival and disease-free survival among high-risk nonmetastatic prostate cancer, when added to the standard treatment of radiotherapy and long-term androgen suppression. Rosenthal et al. (2019) showed a reduction in the rate of distant metastasis with the addition of docetaxel to standard treatment in men [62].

Another WES data application explored was CNA analysis. We found a high-level gain of a chromosomal segment in some CTCs (Figure 8). In the total of nine patients analyzed, four of them had the highest number of amplifications found in different chromosomes (patients 1, 3, 6, and 8). Due to the absence of studies using WES to investigate CNAs in single CTCs from localized high-risk prostate cancer patients before treatment, we compared our finding with those of Friedlander et al. (2019) [52]. The authors performed single-cell whole-genome analysis in CTCs of 14 patients with localized high-risk prostate cancer within 2 to 5 months after prostatectomy. We found amplification in 33 similar genes (Figure 8). As observed by Friedlander et al. (2019) and corroborated by our study, *MYCN* and *AR* amplications was not frequenty observed in CTCs from localized high-risk prostate cancer. None of our CTC-shared CNAs, represented in Figure 8, were observed by Friedlander et al. (2019). In order to investigate which pathways the CTC-shared CNAs (37 genes) could affect, we performed Gene Set Enrichment Analysis (GSEA). PARP1 amplification can affect two important DNA damage repair (DDR) pathways. DDR gene amplification can lead to chemotherapy resistance and short overall survival [53]. PARP1 is a multifunctional enzyme, which binds to DNA breaks and recruits DNA repair proteins to the damaged site [63]. The use of PARP inhibitors in cancer treatment is based on the combination of PARP inhibition with DNA-damaging drugs [63]. Four of the PARP inhibitors are currently approved by FDA for ovarian and breast cancer. However, only a few early phase studies have been completed to propose the use of PARP inhibitors for prostate cancer treatment [63]. A high proportion of prostate cancer patients carry DDR gene defects. Here, we found a higher frequency of amplification on DDR genes as a novel finding of our study. Copy number amplification of DNA damage repair pathways potentiates therapeutic resistance in cancer [63]. These patients may represent a new subgroup that would benefit from therapeutics targeting DNA damage response pathways, such as PARP inhibitors [63]. In addition, USPs (ubiquitin-specific protease) amplification has been reported in prostate cancer, such as USP2a, USP7, and USP10. We showed that USP21 is also amplified in prostate cancer CTCs. USP21 amplification can increase proliferation, migration, and invasion [64]. In non-small-cell lung cancer (NSCLC), USP21 amplification is highly prevalent and it is speculated that inhibition of USP21 might serve as a promising therapeutic approach in NSCLC [64].

The nuclear volume measurements identified two subpopulations: Subpopulation 1 (including patients 3, 4, 5, 6, and 7) and subpopulation 2 (including patients 1, 2, 8, 9, and 10). We found 153 genes commonly affected by missense SNV or frameshift indels in the subpopulation 1 but not in the subpopulation 2. Supplementary Materials Figure S3 shows a heat map in clustered grouping order and a list of all 153 genes found. To date, no study appeared in the literature investigating the association between smaller or larger CTCs with prognosis using CTCs from localized high-risk prostate cancer. In breast cancer patients, for example, smaller CTCs were associated with poor overall survival [65] and the authors suggested that smaller isolated CTCs could be cancer stem cells, and the more cancer stem cells, the more aggressive the disease. For the CNA analysis, we found just one gene commonly amplified in subpopulation 1 (patients 3, 5, 6, and 7) that was not amplified in subpopulation 2 (patients 1, 2, 8, 9, and 10), which was the *MUC12* gene. MUC12 overexpression is an independent marker of prognosis in stage II and stage III colorectal cancer. However, the role of MUC12 overexpression in prostate cancer has not been explored. It is especially important in cancer to distinguish driver mutations from passenger mutations, i.e., to distinguish meaningful events from random background aberrations. Control-FREEC software (version 6.7) identifies those regions of the genome that are aberrant more often than would be expected by chance, with greater weight given to high-amplitude events (high-level copy-number gains or homozygous deletions), which are less likely to represent random aberrations or sequencing errors, and filters for recurrent CNVs that exceed a significance probability threshold of 0.01 [44]. The frequencies of the altered CNAs and SNVs/indels in each group were compared between the subpopulations. A chi square was used to evaluate the statistical significance of the differences. The amplification of the MUC12 gene, which was described in the four patients in group A but not in any of group B, was statistically significant between subpopulations (*p*-value = 6.198 × 10^−12^). The same chi-square test showed a statistically significant difference (*p*-value = 2.2 × 10^−16^) between the pattern of SNVs and indels of the same two subpopulations.

In conclusion, single-cell approaches (WES and 3-D telomere profiling) were shown to be useful in unmasking CTC heterogeneity in a treatment-naïve prostate cancer patient risk group. Tumor heterogeneity is one of the major causes of failure in prostate cancer prognosis and prediction. Accurately detecting tumor heterogeneity and resistant clones is one of the main goals for the identification of new biomarkers for clinical assessment. DDR pathway mutations have been well-established as a target pathway for cancer therapy. However, frequent CNA amplifications found in localized high-risk patients may play critical roles in the therapeutic resistance in prostate cancer. Hence, the single-cell profiling techniques described here, together with other clinical parameters, may aid in the classification of prostate cancer patients and contribute to understanding the predictive value alluded to the presence of genetic alterations, such as SNVs, indels, and CNAs in CTCs subclones.

## Figures and Tables

**Table 1:** List of the 25 samples sequenced: 21 CTCs and 4 lymphocytes.

Sample Name	Patient Number	Patient ID	Type
1011	1	806	CTC
2012	1	806	CTC
3013	2	810	CTC
5015	3	823	CTC
6016	3	823	CTC
7017	3	823	CTC
862_1	5	862	CTC
862_2	5	862	CTC
877_1A	6	877	CTC
877_1B	6	877	CTC
877_1C	6	877	CTC
890_629_3	7	890	CTC
890_2	7	890	CTC
902_815_1	8	902	CTC
902_2	8	902	CTC
902_9	8	902	CTC
922_1	9	922	CTC
922_4	9	922	CTC
964_12	10	964	CTC
964_15	10	964	CTC
964_6	10	964	CTC
922_L	9	922	Lymphocyte
877_3_L	6	877	Lymphocyte
890_L	7	890	Lymphocyte
854_L	4	854	Lymphocyte

**Table 2:** Total number of unique, coding, and non-coding regions affected by single nucleotide variants (SNVs) and insertion-deletions (indels).

	Unique Total	Non-Coding Regions	Coding Regions
SNVs	202,241	192,129 (95%)	10,112 (5%)
Indels	137,407	127,789 (93%)	9618 (7%)

**Table 3:** Single nucleotide variants (SNVs) and insertion-deletions (Indels) count by CTCs and annotation into dbSNP [43] and COSMIC [50] databases.

	SNVs			INDELs		
CTC Sample	Total	Unannotated	Annotated	Total	Unannotated	Annotated
1011 (P1)	82,697	1034	81,663	35,204	773	34,431
2012 (P1)	82,469	952	81,517	36,558	744	35,814
3013 (P2)	96,223	1118	95,105	41,631	971	40,660
5015 (P3)	43,373	1254	42,119	32,658	13,175	19,483
6016 (P3)	47,747	1054	46,693	38,159	14,555	23,604
7017 (P3)	46,047	1139	44,908	37,729	15,468	22,261
862_1 (P5)	44,785	653	44,132	32,845	4309	28,536
862_2 (P5)	51,085	1222	49,863	45,574	16,026	29,548
877_1A (P6)	81,783	1420	80,363	49,401	12,387	37,014
877_1B (P6)	5052	193	4859	4261	1607	2654
877_1C (P6)	16,818	229	16,589	12,077	1775	10,302
890_629_3 (P7)	21,506	655	20,851	19,648	6462	13,186
890_2 (P7)	56,421	1370	55,051	43,329	16,340	26,989
902_815_1 (P8)	36,729	532	36,197	19,511	2108	17,403
902_2 (P8)	8380	338	8042	7789	2988	4801
902_9 (P8)	27,091	1042	26,049	27,608	11,530	16,078
922_1 (P9)	4503	276	4227	9596	3913	5683
922_4 (P9)	386	154	232	596	525	71
964_12 (P10)	5441	332	5109	4790	2229	2561
964_15 (P10)	16,671	539	16,132	13,049	4649	8400
964_6 (P10)	7500	393	7107	10,771	3844	6927

Unannotated: not found in both database (dbSNP and COSMIC). Annotated: found in at least one database (dbSNP and/or COSMIC). P = Patient.

**Table 4:** Gene set enrichment analysis of the 37 shared amplified genes using the Reactome database found in patients 1, 3, 6, and 8.

Term	*p*-Value	Genes
Generic Transcription Pathway Homo sapiens R-HSA-212436	6.28 × 10^−4^	ZFP14; ZNF461; PARP1; ZNF382; ZNF529; ZNF566; TEAD1
POLB-Dependent Long Patch Base Excision Repair Homo sapiens R-HSA-110362	0.01288	PARP1
Regulation of cytoskeletal remodeling and cell spreading by IPP complex components Homo sapiens R-HSA-446388	0.014707	PARVA
HDR through MMEJ (alt-NHEJ) Homo sapiens R-HSA-5685939	0.018351	PARP1
Dectin-2 family Homo sapiens R-HSA-5621480	0.018351	FCER1G
PPARA activates gene expression Homo sapiens R-HSA-1989781	0.018526	APOA2;TEAD1
Regulation of lipid metabolism by Peroxisome proliferator-activated receptor alpha (PPARalpha) Homo sapiens R-HSA-400206	0.01946	APOA2;TEAD1
Heme biosynthesis Homo sapiens R-HSA-189451	0.020168	PPOX
Serotonin receptors Homo sapiens R-HSA-390666	0.021981	HTR4
Platelet Adhesion to exposed collagen Homo sapiens R-HSA-75892	0.023792	FCER1G
TNFR1-induced proapoptotic signaling Homo sapiens R-HSA-5357786	0.023792	USP21

## Supplementary Materials

The following are available online at https://www.mdpi.com/2073-4409/9/8/1863/s1, Figure S1: Representative bar plots to illustrate the lymphocytes telomere parameters. Figure S2: Venn diagram representing CTC-shared SNVs/indels and CNA per patient. Genes highlight in the CNA section were also found in Friedlander et al. 2019 and genes highlight in the SNVs/indels were associated with prostate cancer in bold and telomere maintenance in italic. We also emphasize in red CTC-shared genes. Figure S3: A heat map of gene alterations found in two subpopulations identified by nuclear volume measurements. Table S1: List of all patients included with number of CTCs and/or lymphocytes analyzed, corresponding Gleason score, TMN staging and PSA levels at diagnosis. Table S2: SNVs associated with known cancer drugs in PharmGKB database. Table S3: List of 37 commonly amplified-genes for patients 1, 3, 6 and 8.
