# Supplementary material for: Genomic Analysis of Localized High-Risk Prostate Cancer Circulating Tumor Cells at the Single-Cell Level
Source: Cells. 2020 Aug 8;9(8):1863. doi: 10.3390/cells9081863 (PMC7466090; doi:10.3390/cells9081863)
Supplement: Supplementary file 1 [file cells-09-01863-s001.zip › Supplementary Figure 1.docx]

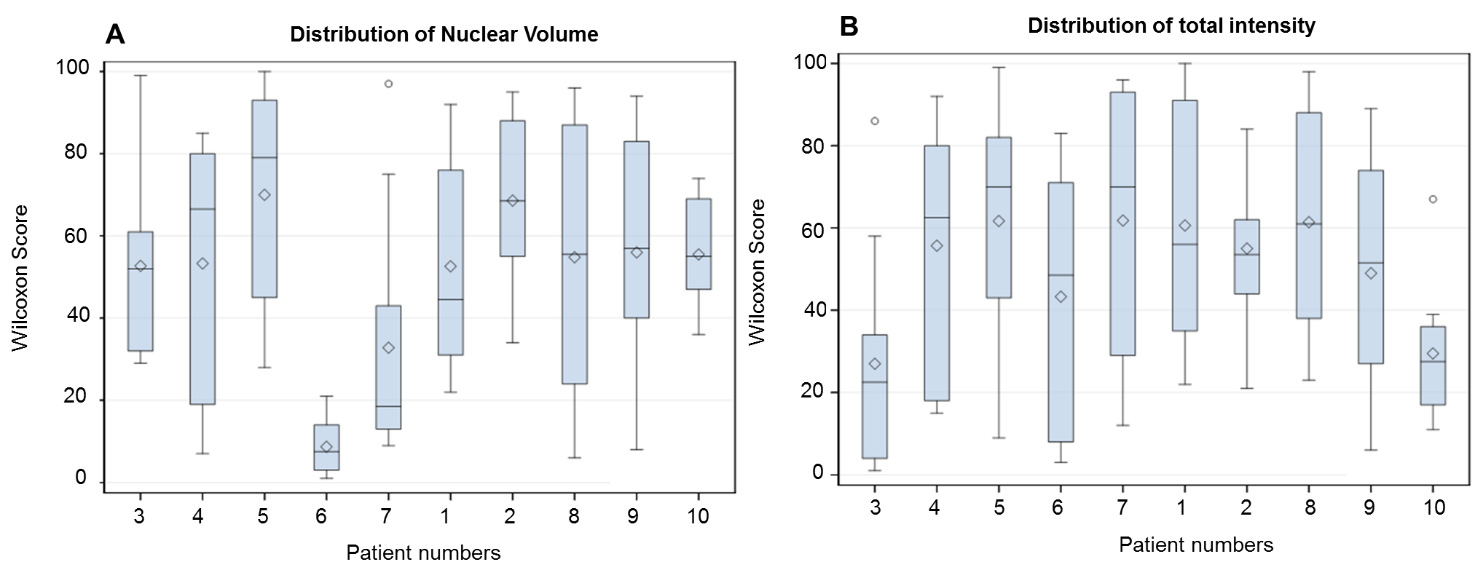


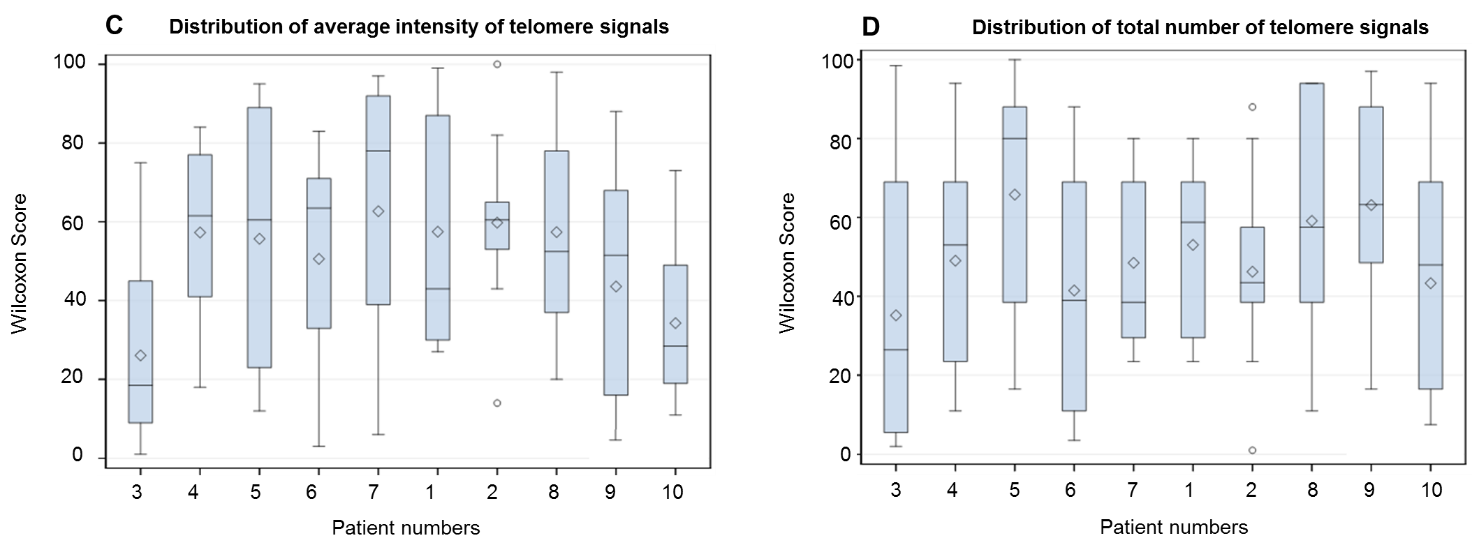


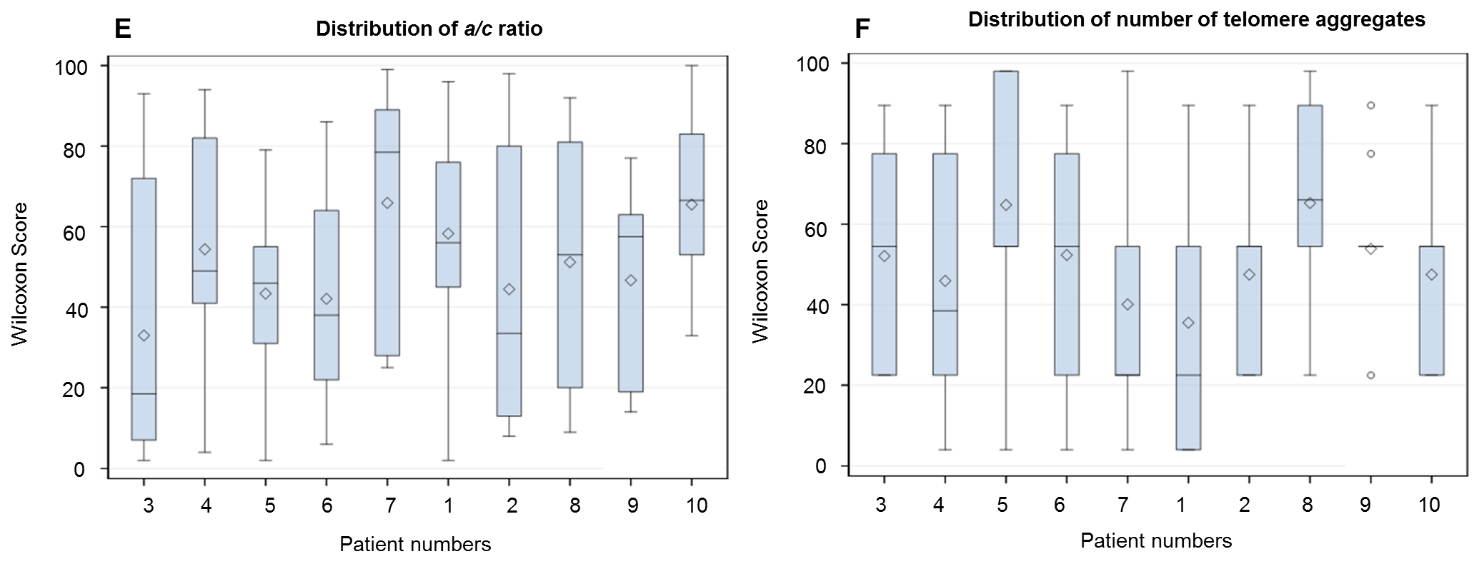


**Supplementary Figure 1**. Representative bar plots to illustrate the lymphocytes telomere parameters. (A) Nuclear volume. (B) Total telomere signal intensity. (C) Average intensity (proportional of telomere length). (D) Total number of telomere signals. (E) a/c ratio (see material and methods). (F) Total number of telomere aggregates (see material and methods). The x-axis assigns one box for the lymphocyte population analyzed per patient. The y-axis refers to output from Kruskal– Wallis test represented as Wilcoxon mean scores (determined using SAS software). Whiskers show minimum and maximum values, boxes represent 25–75% data ranges, horizontal lines within boxes are medians, and diamond symbols are means.
