# Supplementary material for: Genomic Analysis of Localized High-Risk Prostate Cancer Circulating Tumor Cells at the Single-Cell Level
Source: Cells. 2020 Aug 8;9(8):1863. doi: 10.3390/cells9081863 (PMC7466090; doi:10.3390/cells9081863)
Supplement: Supplementary file 1 [file cells-09-01863-s001.zip › Supplementary Figure 2.pdf]

Supplementary Figures 2: Venn diagram representing CTC-shared SNVs/indels and CNA per patient. Genes highlight in the CNA section were also found in Friedlander *et al.* 2019 and genes highlight in the SNVs/indels were associated with prostate cancer in bold and telomere maintenance in italic. We also emphasize in red CTC-shared genes.

Copy Number Alterations (CNA)

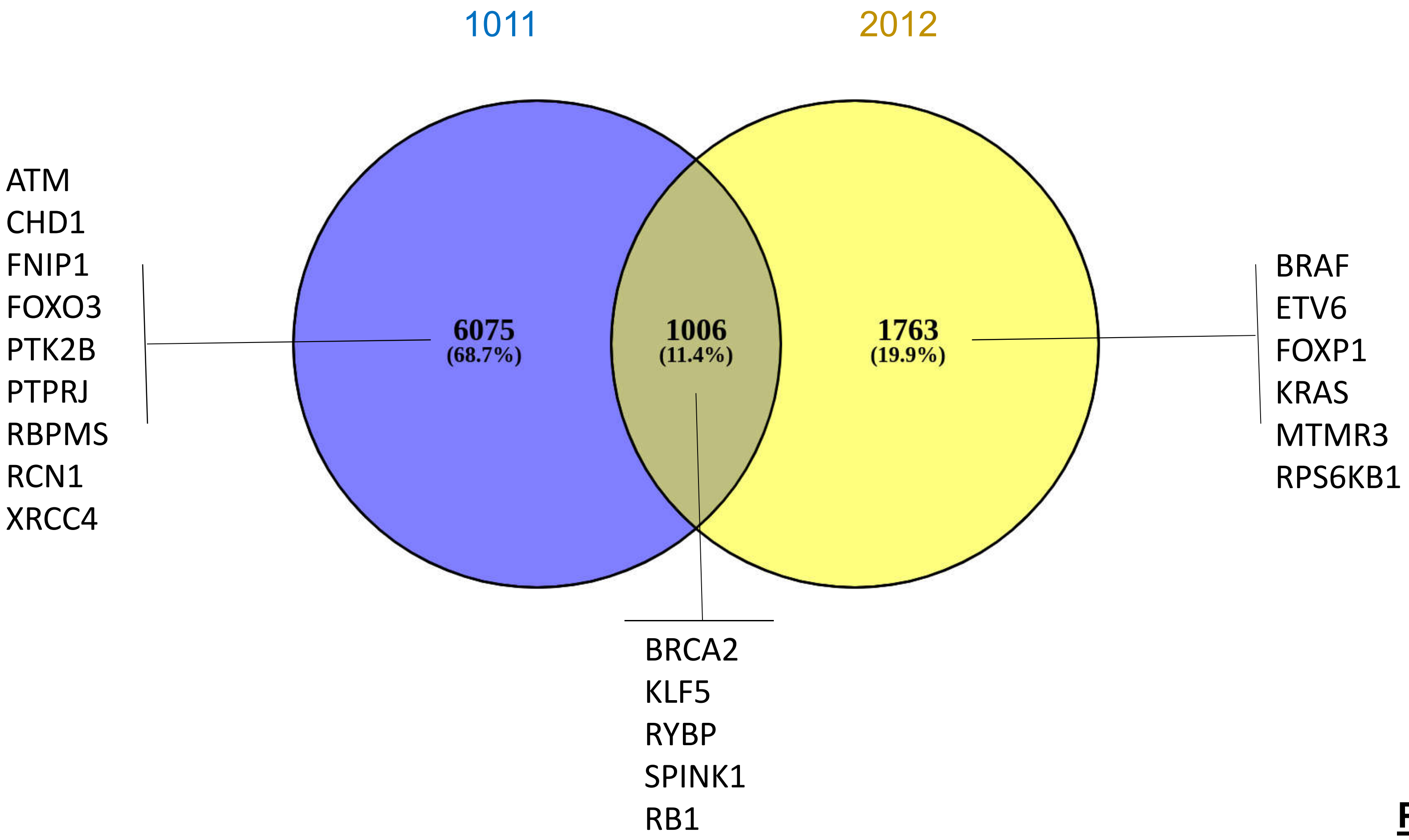
1011
2012
6075
(68.7%)
1006
(11.4%)
1763
(19.9%)
ATM
CHD1
FNIP1
FOXO3
PTK2B
PTPRJ
RBPMS
RCN1
XRCC4
BRAF
ETV6
FOXP1
KRAS
MTMR3
RPS6KB1
BRCA2
KLF5
RYBP
SPINK1
RB1

Patient 1

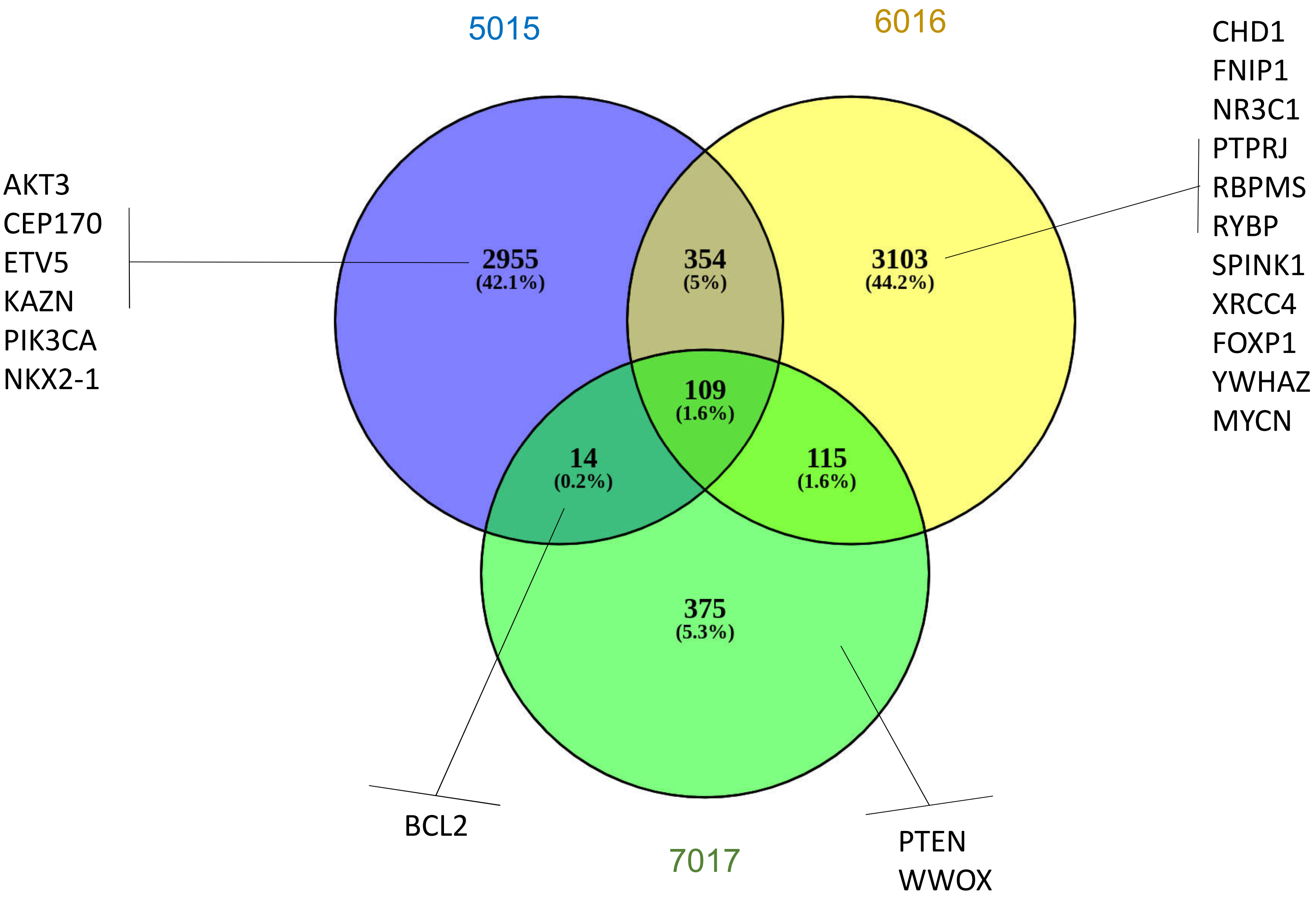
5015
6016
7017
2955
(42.1%)
354
(5%)
3103
(44.2%)
109
(1.6%)
14
(0.2%)
115
(1.6%)
375
(5.3%)
AKT3
CEP170
ETV5
KAZN
PIK3CA
NKX2-1
CHD1
FNIP1
NR3C1
PTPRJ
RBPMS
RYBP
SPINK1
XRCC4
FOXP1
YWHAZ
MYCN
BCL2
PTEN
WWOX

Patient 3

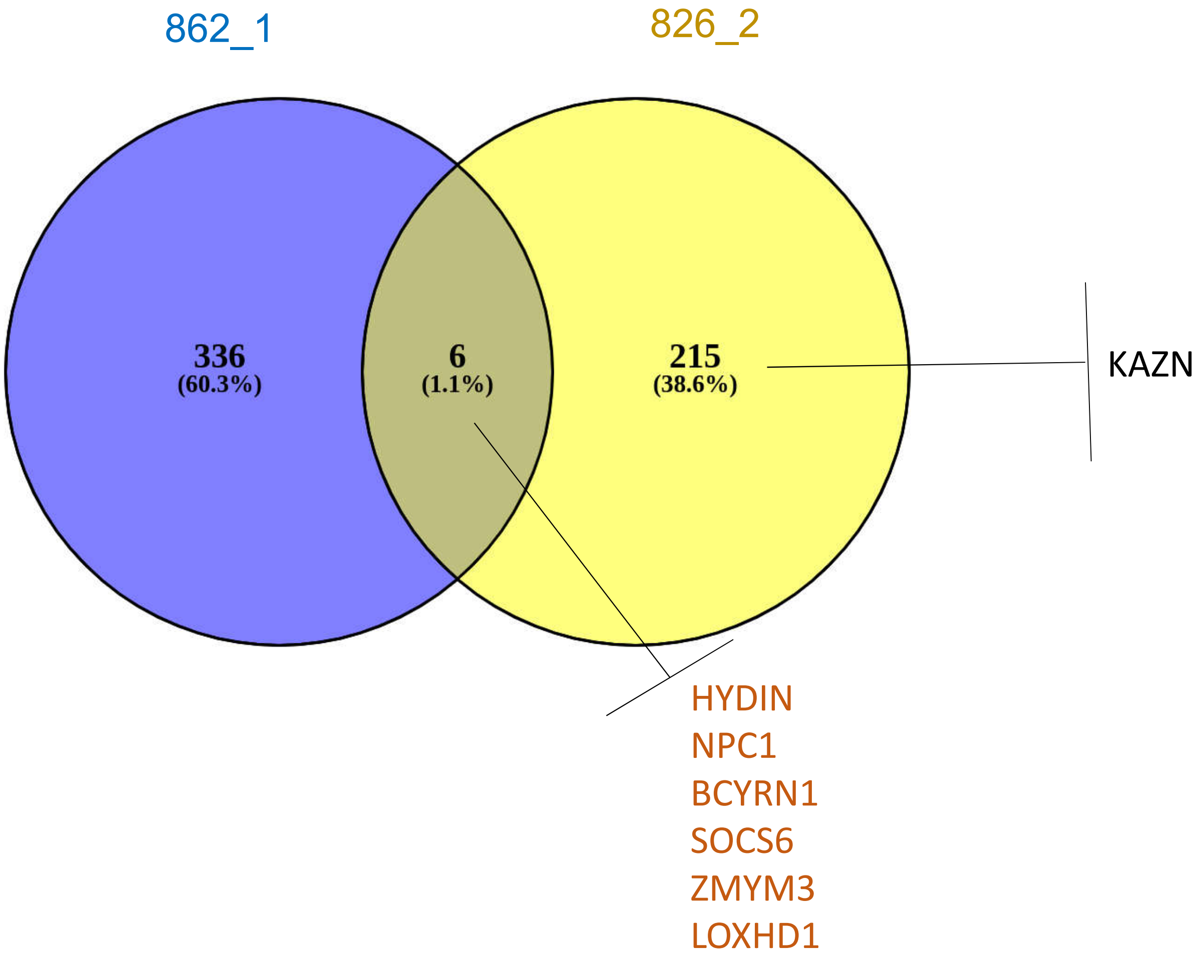
862_1
826_2
336
(60.3%)
6
(1.1%)
215
(38.6%)
KAZN
HYDIN
NPC1
BCYRN1
SOCS6
ZMYM3
LOXHD1

Patient 5

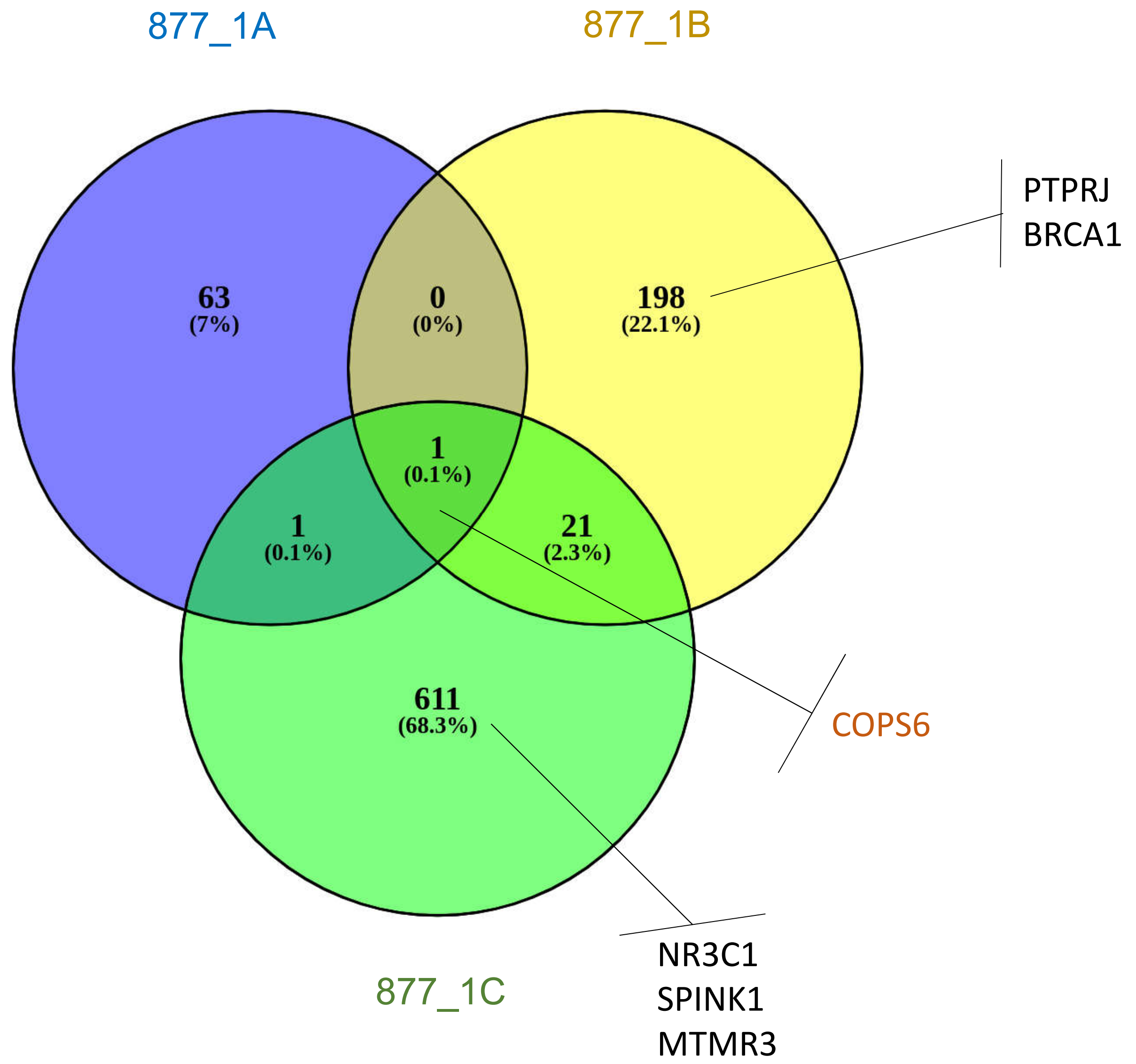

877_1A
877_1B
877_1C
63
(7%)
0
(0%)
198
(22.1%)
1
(0.1%)
1
(0.1%)
21
(2.3%)
611
(68.3%)
PTPRJ
BRCA1
COPS6
NR3C1
SPINK1
MTMR3

Patient 6

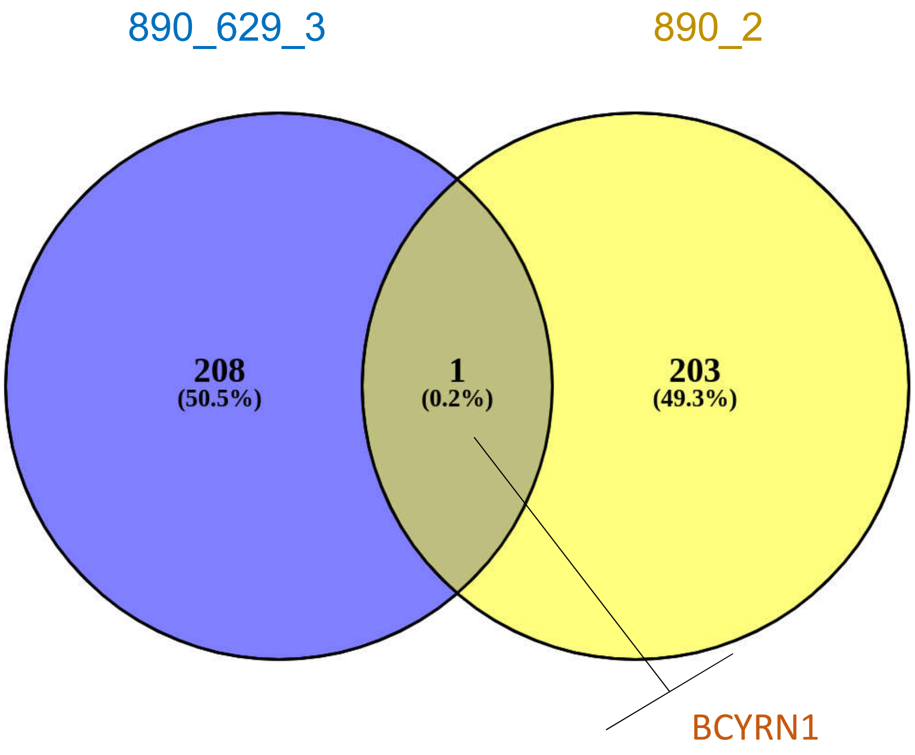

Patient 7

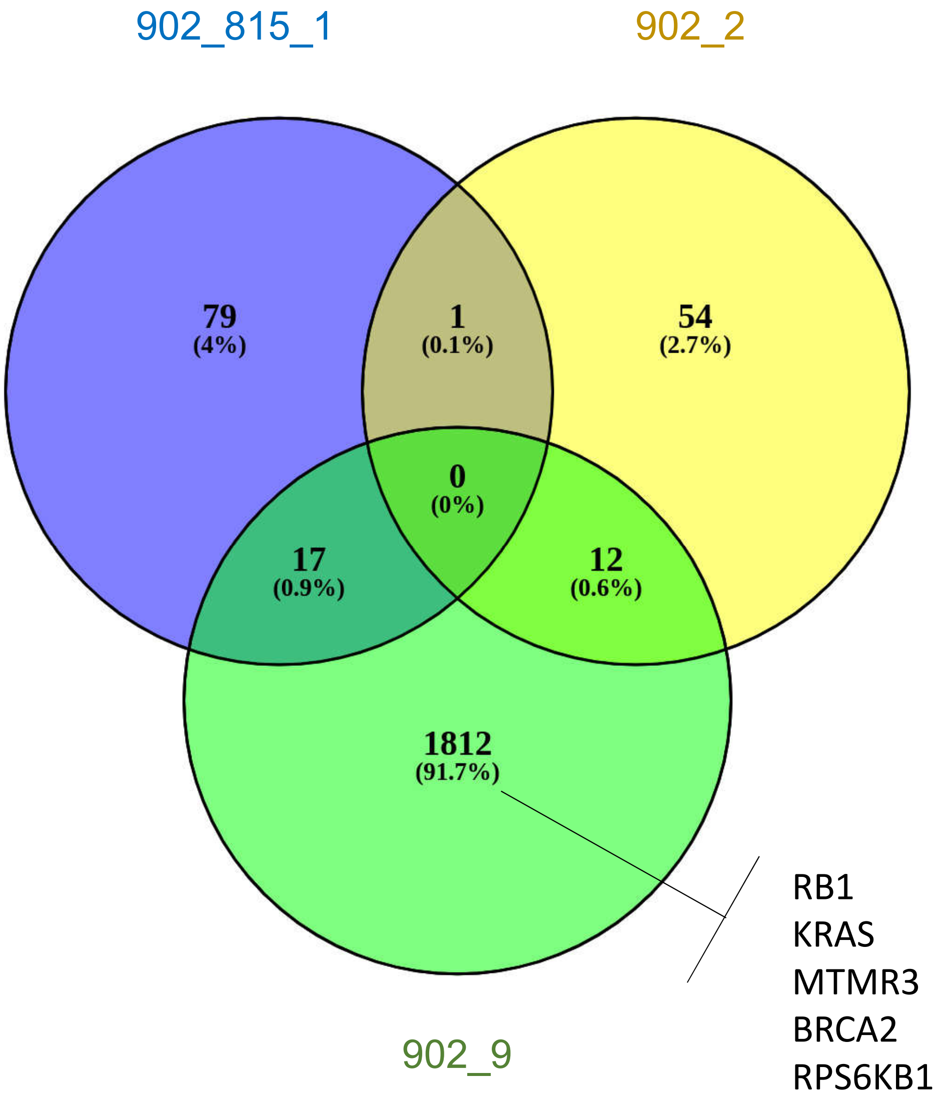
902_815_1
902_2
902_9
79
(4%)
1
(0.1%)
54
(2.7%)
0
(0%)
17
(0.9%)
12
(0.6%)
1812
(91.7%)
RB1
KRAS
MTMR3
BRCA2
RPS6KB1

Patient 8

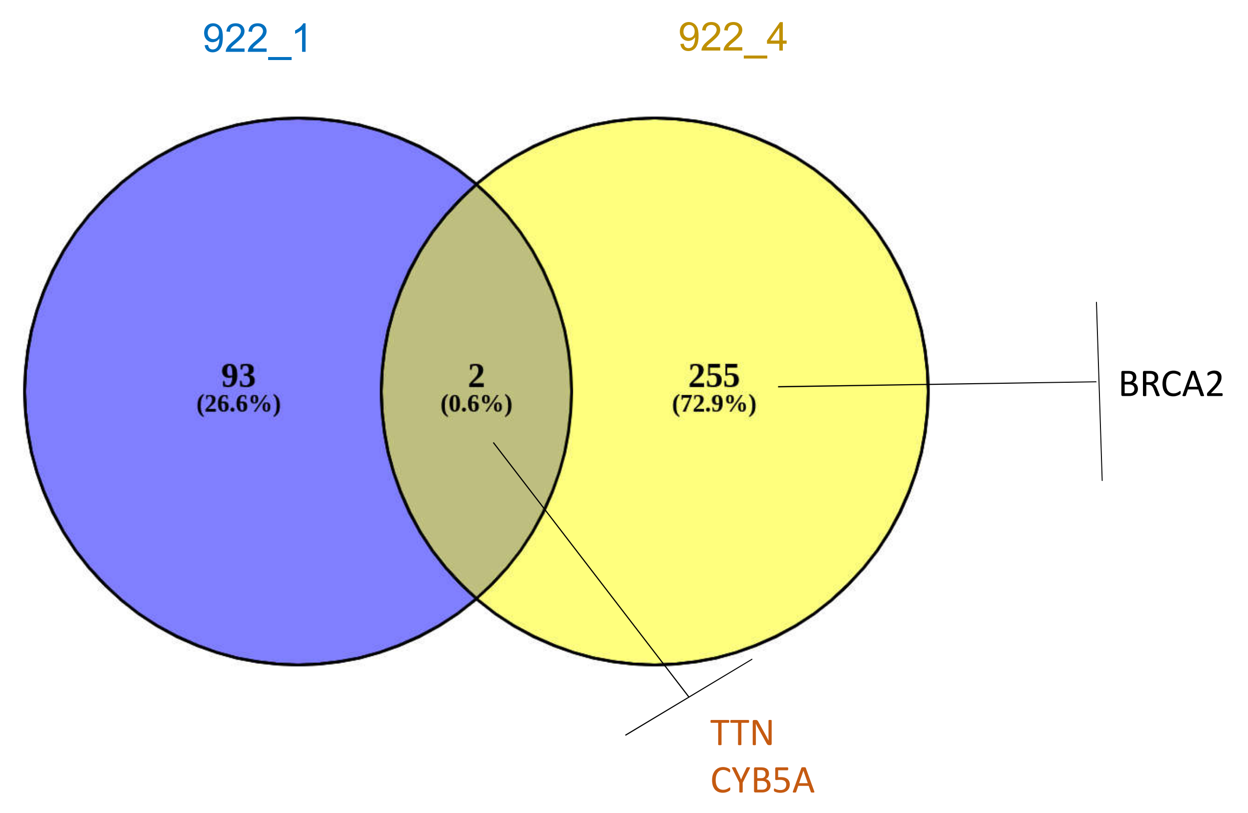

Patient 9

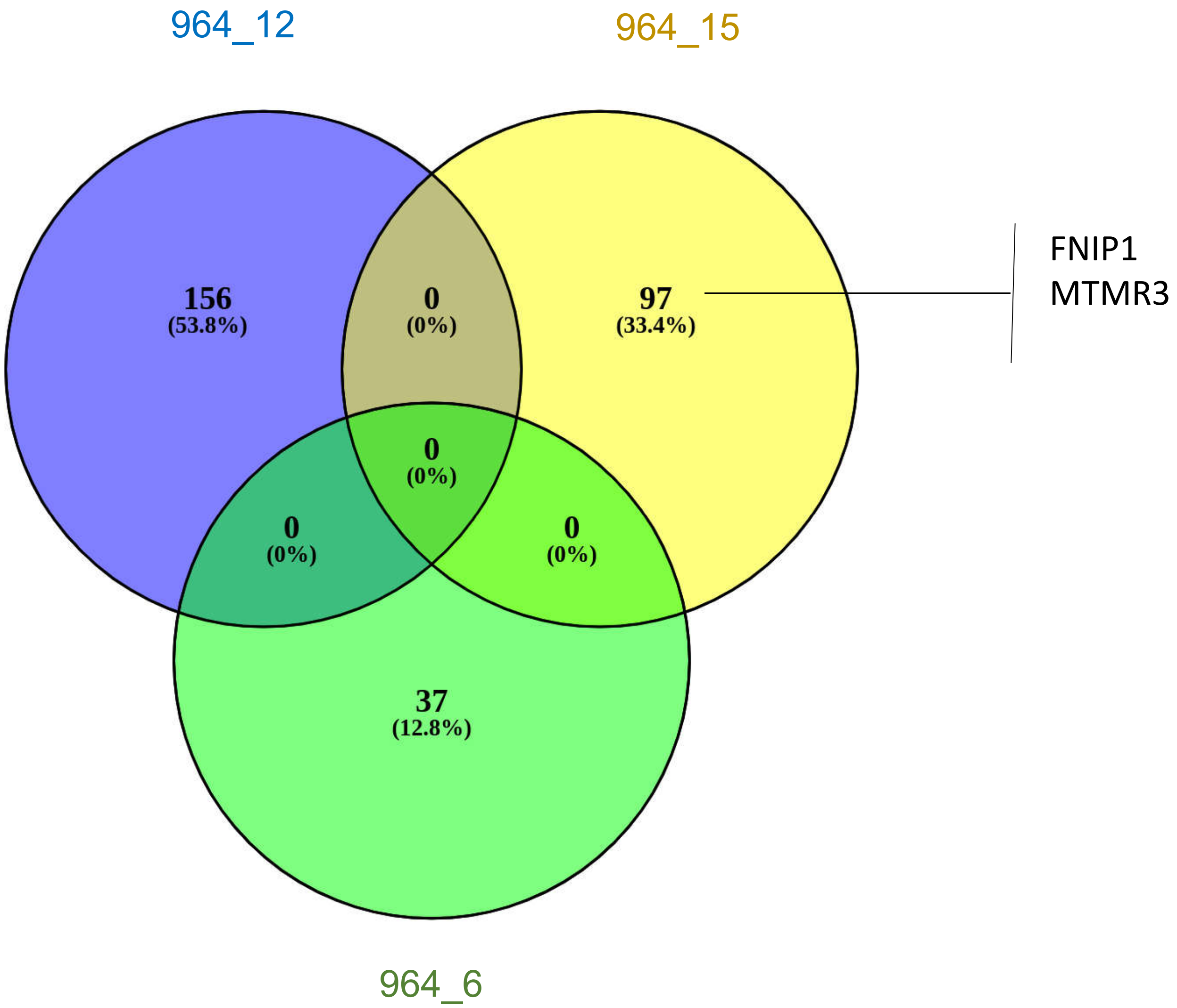

Patient 10

Genetic variations (SNP + INDELs)

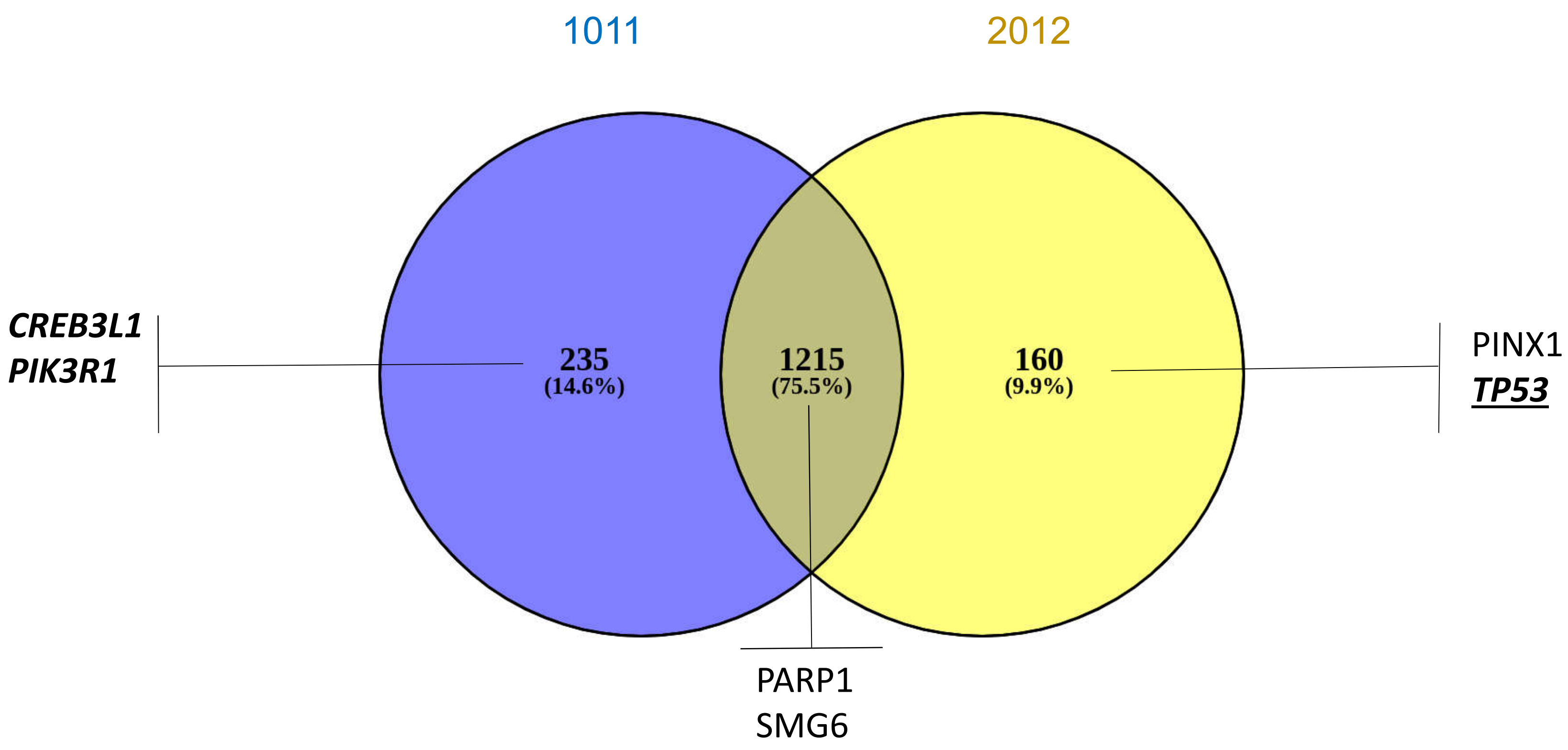
1011
2012
CREB3L1
PIK3R1
235
(14.6%)
1215
(75.5%)
160
(9.9%)
PINX1
TP53
PARP1
SMG6

Patient 1

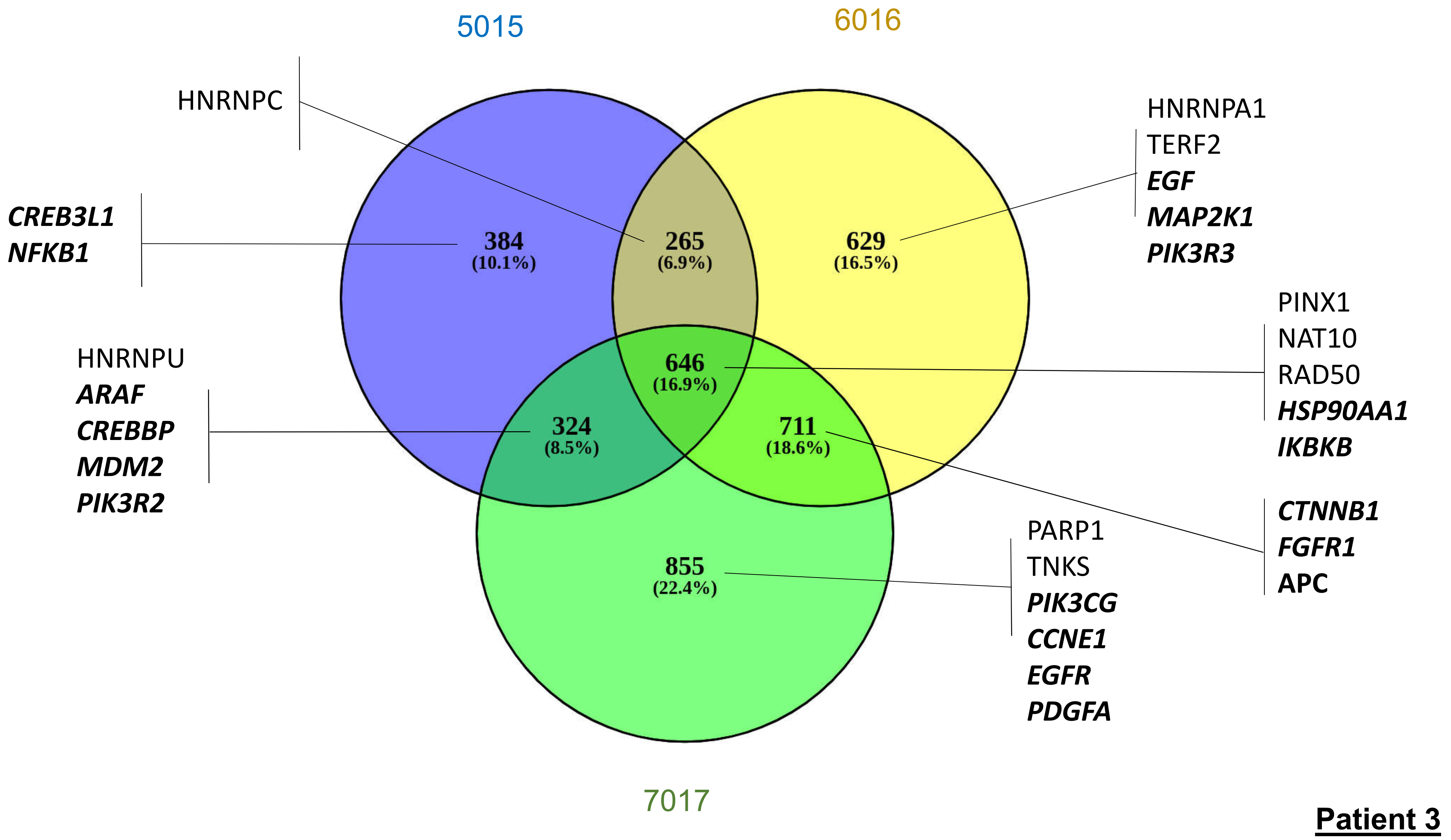
5015
6016
7017
HNRNPC
CREB3L1
NFKB1
384
(10.1%)
265
(6.9%)
629
(16.5%)
HNRNPA1
TERF2
EGF
MAP2K1
PIK3R3
PINX1
NAT10
RAD50
HSP90AA1
IKBKB
646
(16.9%)
HNRNPU
ARAF
CREBBP
MDM2
PIK3R2
324
(8.5%)
711
(18.6%)
CTNNB1
FGFR1
APC
855
(22.4%)
PARP1
TNKS
PIK3CG
CCNE1
EGFR
PDGFA
Patient 3

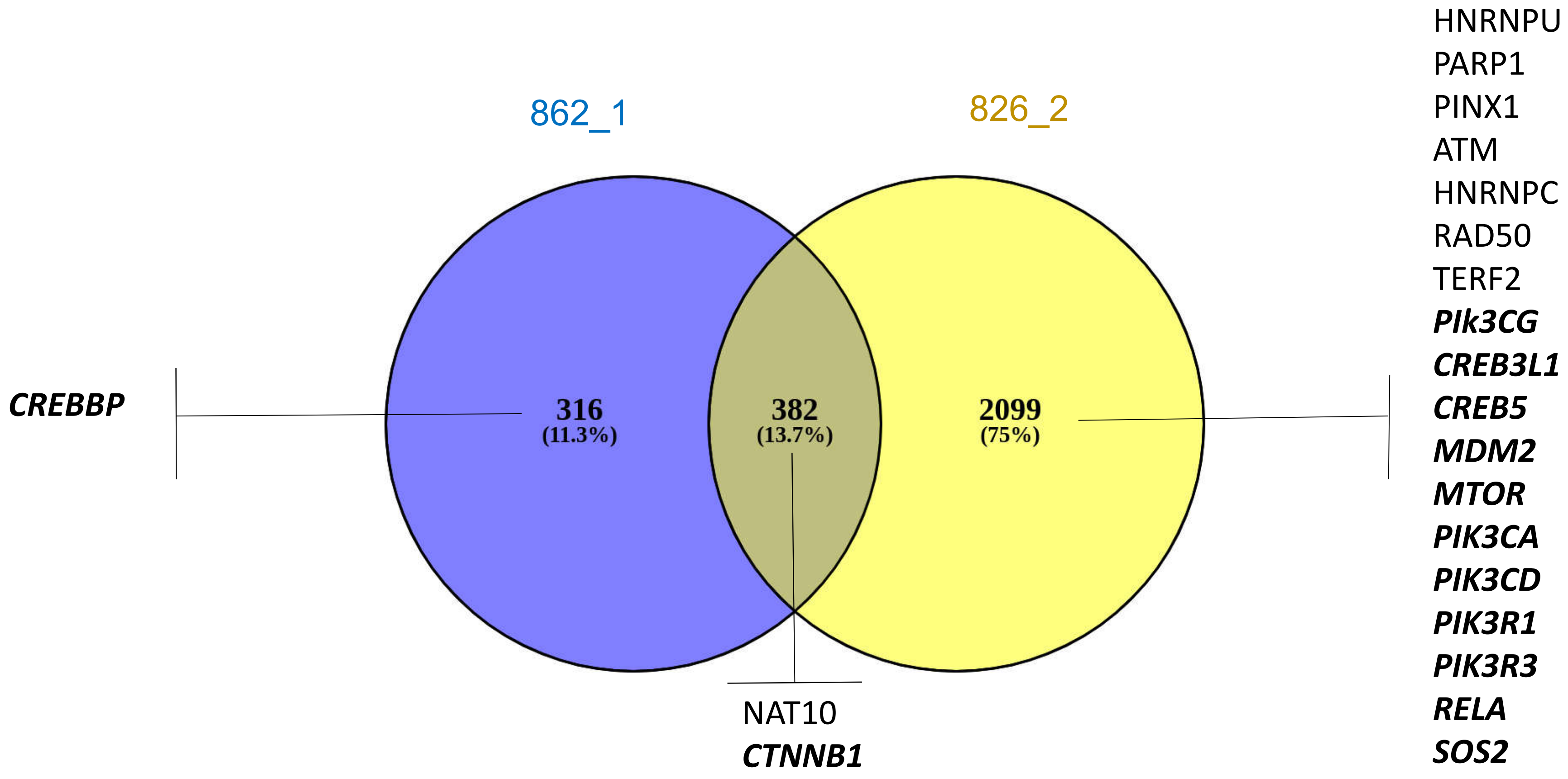
862_1
826_2
CREBBP
316
(11.3%)
382
(13.7%)
2099
(75%)
NAT10
CTNNB1
HNRNPU
PARP1
PINX1
ATM
HNRNPC
RAD50
TERF2
PIk3CG
CREB3L1
CREB5
MDM2
MTOR
PIK3CA
PIK3CD
PIK3R1
PIK3R3
RELA
SOS2
TP53
APC

Patient 5

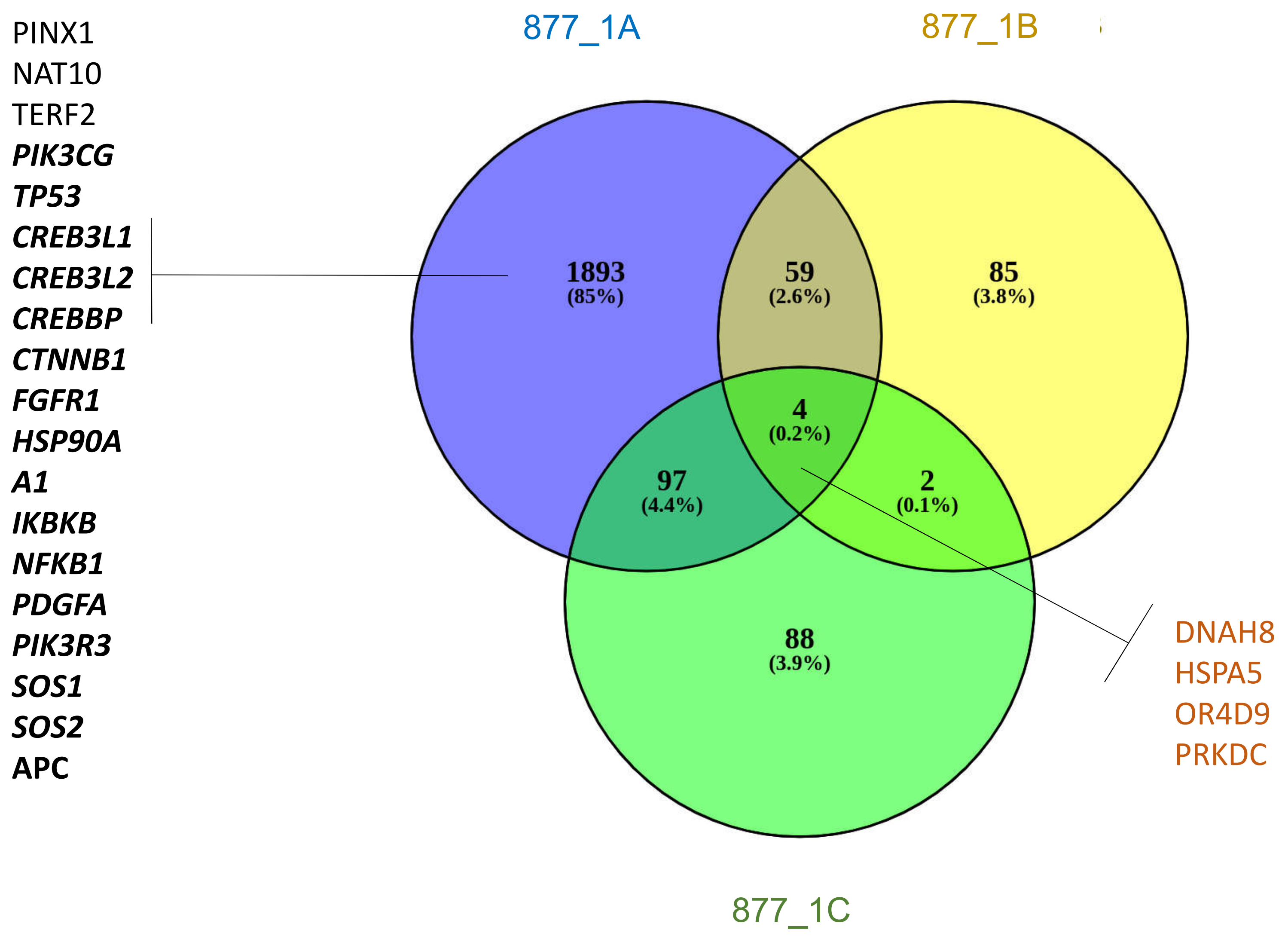
PINX1
NAT10
TERF2
PIK3CG
TP53
CREB3L1
CREB3L2
CREBBP
CTNNB1
FGFR1
HSP90A
A1
IKBKB
NFKB1
PDGFA
PIK3R3
SOS1
SOS2
APC
877_1A
877_1B
1893
(85%)
59
(2.6%)
85
(3.8%)
4
(0.2%)
97
(4.4%)
2
(0.1%)
88
(3.9%)
DNAH8
HSPA5
OR4D9
PRKDC
877_1C

Patient 6

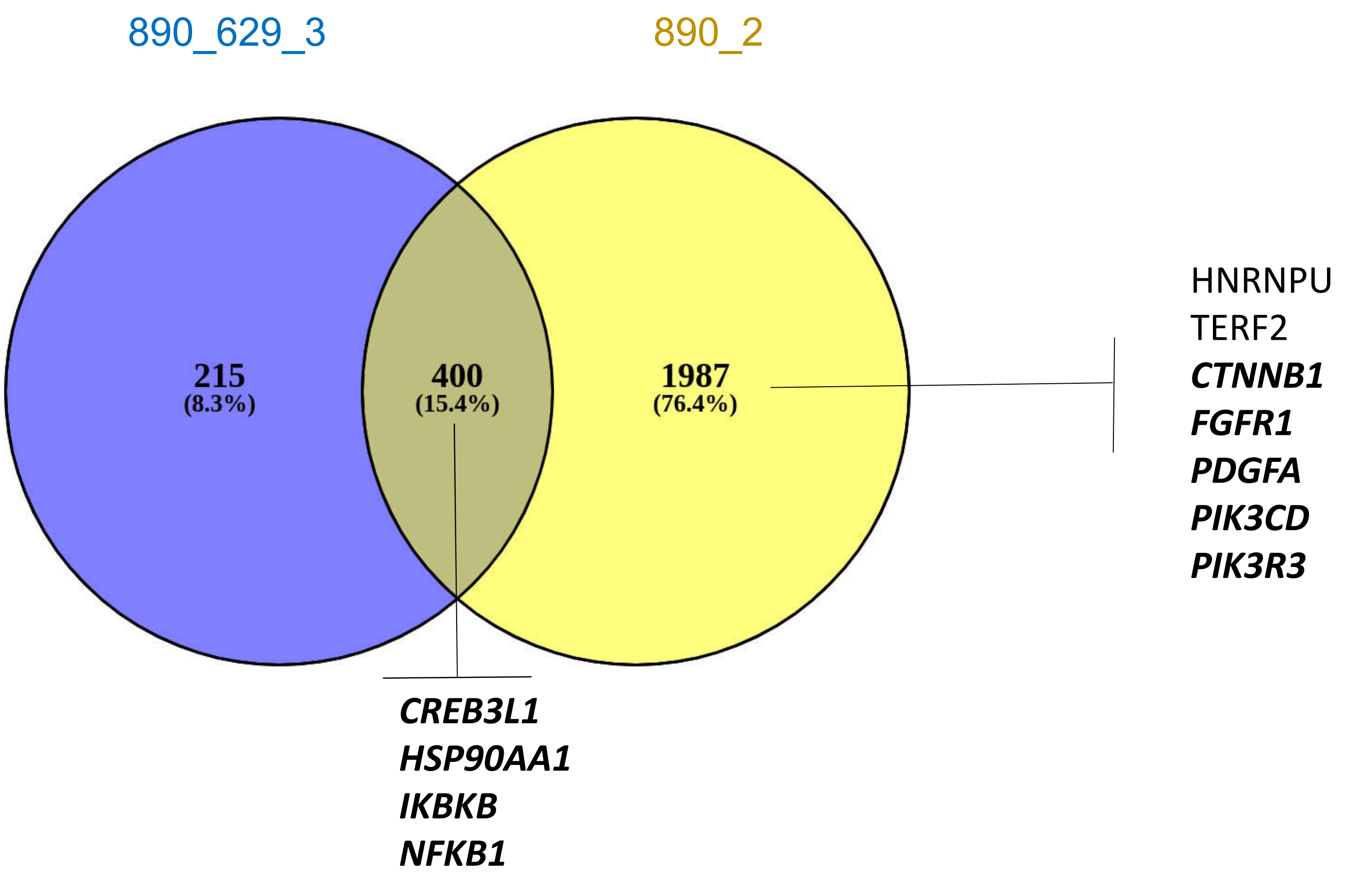
890_629_3
890_2
215
(8.3%)
400
(15.4%)
1987
(76.4%)
HNRNPU
TERF2
CTNNB1
FGFR1
PDGFA
PIK3CD
PIK3R3
CREB3L1
HSP90AA1
IKBKB
NFKB1

Patient 7

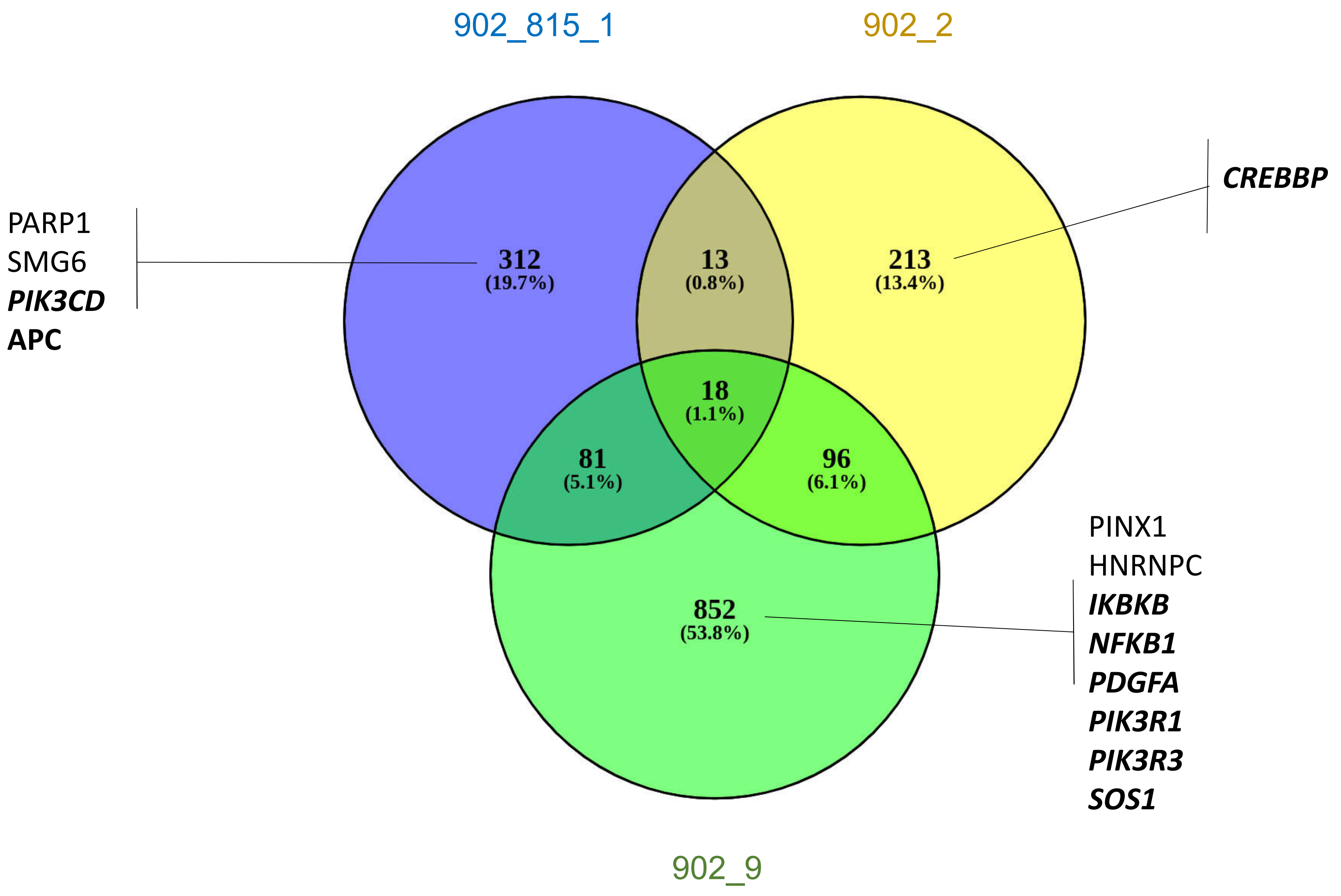
902_815_1
902_2
PARP1
SMG6
PIK3CD
APC
312
(19.7%)
13
(0.8%)
213
(13.4%)
CREBBP
18
(1.1%)
81
(5.1%)
96
(6.1%)
852
(53.8%)
PINX1
HNRNPC
IKBKB
NFKB1
PDGFA
PIK3R1
PIK3R3
SOS1
902_9

Patient 8

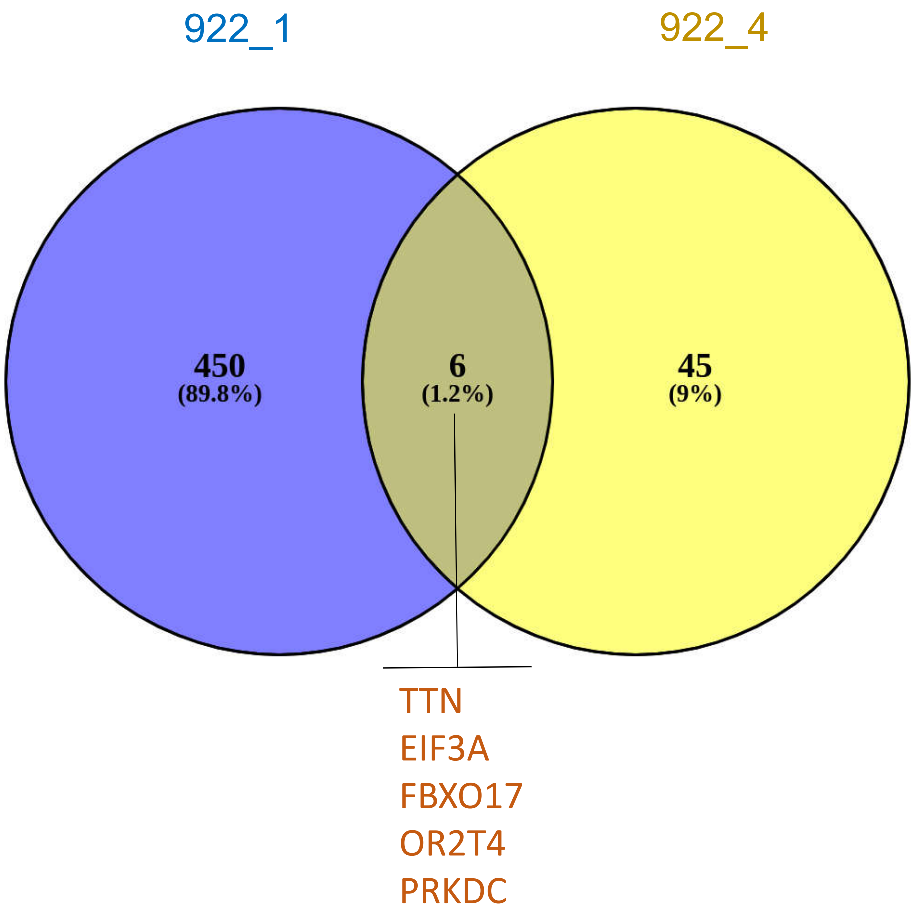

Patient 9

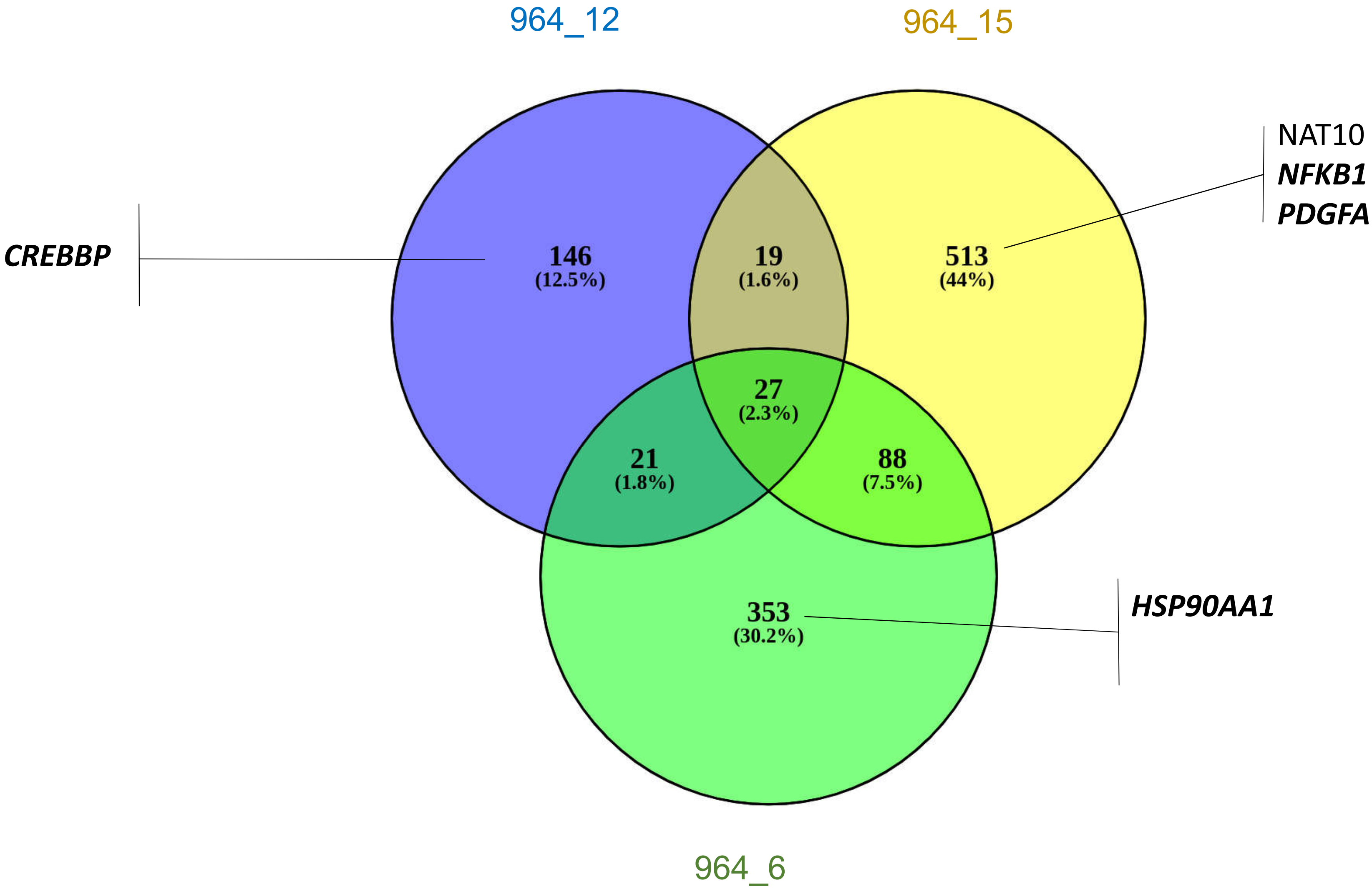
964_12
964_15
964_6
CREBBP
NAT10
NFKB1
PDGFA
HSP90AA1
146
(12.5%)
19
(1.6%)
513
(44%)
27
(2.3%)
21
(1.8%)
88
(7.5%)
353
(30.2%)

Patient 10
