# Supplementary material for: Genomic Analysis of Localized High-Risk Prostate Cancer Circulating Tumor Cells at the Single-Cell Level
Source: Cells. 2020 Aug 8;9(8):1863. doi: 10.3390/cells9081863 (PMC7466090; doi:10.3390/cells9081863)
Supplement: Supplementary file 1 [file cells-09-01863-s001.zip › Supplementary figure 3.docx]

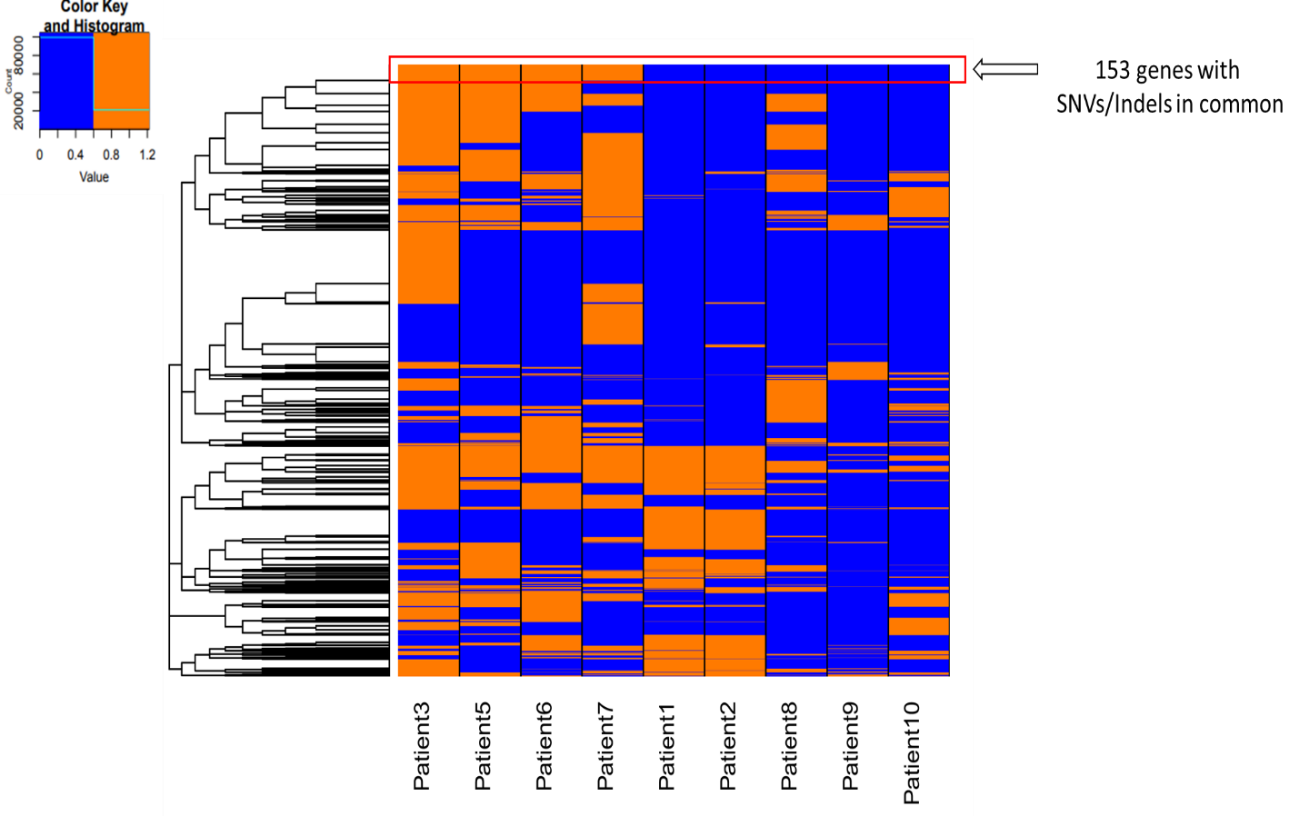


**Supplementary figure 3A**. A heat map of gene alterations found in two subpopulations identified by nuclear volume measurements. All genes affected were listed in a heat map in clustered grouping order. Genes with the missense SNV or frameshift Indels alterations, shown in whole exome sequencing, were marked in orange, and genes with no alterations were shown in blue. Differences between the two groups are highlighted in the box in red. SNV: single nucleotide variation; Indel: Insertion and deletion mutations.

**Supplementary figure 3B**: 153 genes commonly affected by missense SNV or frameshift indels in subpopulation 1, absent in subpopulation 2.


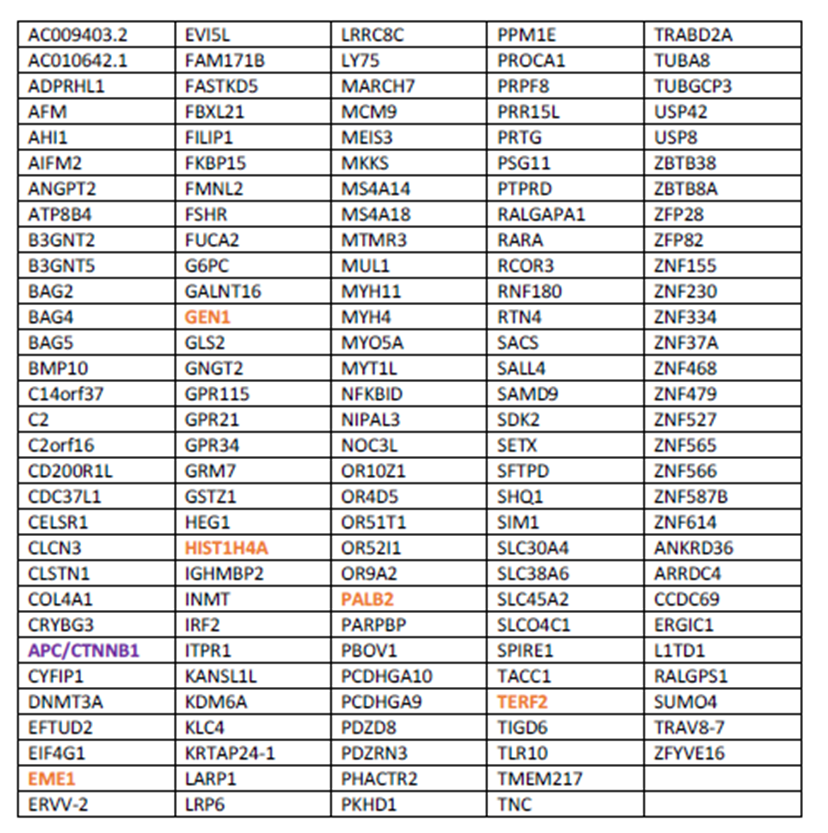


The genes in orange are associated with telomere maintenance and the gene in purple with prostate cancer. For those genes, we found an insertion of 2 nucleotides in *APC/CTNNB1* gene (location: rs41275332); insertion of 2 nucleotides in *EME1* gene (rs48453320); insertion of 19 nucleotides in *GEN1* gene (rs17962850); insertion of 1 nucleotide in *PALB2* gene (rs23647203); and insertion of 16 nucleotides in *TERF2* gene (rs69402438).
