# Supplementary material for: Genomic Analysis of Localized High-Risk Prostate Cancer Circulating Tumor Cells at the Single-Cell Level
Source: Cells. 2020 Aug 8;9(8):1863. doi: 10.3390/cells9081863 (PMC7466090; doi:10.3390/cells9081863)
Supplement: Supplementary file 1 [file cells-09-01863-s001.zip › Supplementary Tables.docx]

**Table S1**: List of all patients included with number of CTCs and Lymphocytes analyzed, corresponding Gleason score, TMN staging at baseline, Ethnicity/race, PSA levels at diagnosis and treatment received in the end.

| **Patient** | **Patient ID** | **Grouping** | **Number of CTCs analyzed** | **Number of Lymphocytes analyzed** | **Gleason score** | **TNM staging** | **Ethnicity** | **PSA pre-TRX** |
| --- | --- | --- | --- | --- | --- | --- | --- | --- |
| **1** | 806 | High Risk | 2 | - | 9 | T2c | CA | 5,30 |
| **2** | 810 | High Risk | 1 | - | 8 | T1c | CA | 5,30 |
| **3** | 823 | High Risk | 3 | - | 8 | T3b | CA | 16,20 |
| **4** | 854 | High Risk | - | 1 | 9 | T1c | CA | 1,53 |
| **5** | 862 | High Risk | 2 | - | 9 | T1c | CA | 23,30 |
| **6** | 877 | High Risk | 3 | 1 | 9 | T1c | CA | 5,78 |
| **7** | 890 | High Risk | 2 | 1 | 9 | T1c | CA | 18,00 |
| **8** | 902 | High Risk | 3 | 1 | 9 | T2c | CA | 7,1 |
| **9** | 922 | High Risk | 2 | - | 9 | T1c | CA | 5,83 |
| **10** | 964 | High Risk | 3 | - | 9 | T1c | CA | 8,29 |

CA: Caucasian

**Table S2**: SNVs associated with known cancer drugs in PharmGKB database

| **Drug Name**  **(* associated to prostate cancer)** | **Number of SNVs found associated with this drug response** | **Percentage of all existing SNVs associated with this drug in**  **PharmKGB database** | **Fisher exact test (p-value)** | **FDR (False Discovery Rate)** | **Patients possessing the corresponding SNVs**  **(1 or more)** |
| --- | --- | --- | --- | --- | --- |
| Anthracyclines | 4 | 10.5% | 0.02 | 0.09 | 3, 5, 6, 8 |
| Capecitabine | 5 | 5.5% | 0.50 | 0.86 | 1, 2, 3, 5, 6, 8, 9 |
| Carboplatin | 6 | 11% | 0.25 | 0.50 | 1, 3, 5, 6, 8, 10 |
| Cisplatin | 4 | 2.9% | 0.88 | 1.00 | 1, 2, 3, 6, 7 |
| Cyclophosphamide(*) | 3 | 1.9% | <2.20e-16 | <2.20e-16 | 1, 2, 3, 6, 7 |
| Docetaxel (*) | 9 | 7.9% | <2.20e-16 | <2.20e-16 | 1, 3, 5, 6, 8, 10 |
| Doxorubicin | 3 | 2.9% | 0.13 | 0.36 | 1, 2, 3, 6 |
| doxorubicinol | 1 | 33.3% | 0.21 | 0.46 | 1, 2, 6 |
| Exemestane | 1 | 6.6% | 1 | 1 | 3 |
| Fluorouracil | 8 | 4.3% | 7.47e-2 | 0.27 | 1, 2, 3, 5, 6, 7 |
| Gemcitabine | 6 | 7.9% | 0.02 | 0.09 | 2, 3, 5, 6, 7 |
| Imatinib | 1 | 2.7% | 1 | 1 | 1, 2, 3, 7, 10 |
| Irinotecan | 4 | 4.8% | 0.29 | 0.54 | 1, 6, 8 |
| Letrozole | 1 | 4.7% | 1 | 1 | 3 |
| Leucovorin | 2 | 4.2% | 0.78 | 0.99 | 6 |
| Lonafarnib | 1 | 100% | 0.08 | 0.27 | 5, 6 |
| Methotrexate | 2 | 1.4% | 0.54 | 0.86 | 3, 7 |
| Oxaliplatin | 1 | 1.5% | 0.63 | 0.93 | 5, 6, 7 |
| Paclitaxel | 6 | 8.7% | 0.70 | 0.93 | 1, 2, 3, 5, 6, 8, 10 |
| Platin compounds | 2 | 2.4% | 1 | 1 | 2, 6 |
| Thalidomide (*) | 5 | 19% | 5.09e-4 | 4.07e-3 | 5, 6, 7, 8, 10 |
| Tipiracil HCL | 1 | 50% | 0.15 | 0.36 | 1, 5 |
| Trastuzumab | 1 | 4.5% | 0.68 | 0.93 | 3 |
| Trifluridine | 1 | 50% | 0.15 | 0.36 | 1, 5 |

* Drugs associated with prostate cancer

**Table S3**: List of 37 commonly amplified-genes for patients 1, 3, 6 and 8.

| ADAMTS4 | KLHDC9 | OR5AK2 | SLAMF1 | ZNF260 | HTR4 |
| --- | --- | --- | --- | --- | --- |
| APOA2 | LOC644189 | PARP1 | TEAD1 | ZNF382 | PRR14L |
| ARHGAP30 | LOC728752 | PARVA | TNFSF4 | ZNF461 |  |
| B4GALT3 | MROH9 | PFDN2 | UFC1 | ZNF529 |  |
| DEDD | NDUFS2 | PPOX | USP21 | ZNF566 |  |
| EIF1AY | NECTIN4 | RC3H1 | ZFP14 | DEPDC5 |  |
| FCER1G | NIT1 | RPS4Y2 | ZFP82 | FBXO38 |  |
